# Ciprofloxacin and Purple Carrot Extract-Loaded Bilayer Nanofibrous Wound Dressings: A Multifunctional Platform for Controlled Release and Antibacterial Wound Care

**DOI:** 10.3390/polym18202479

**Published:** 2026-10-10

**Authors:** Petek Nisa Kahraman, Sema Samatya Yilmaz, Huseyin Uzuner, Ikrime Orkan Ucar, Ayse Aytac

**Affiliations:** 1 Department of Chemical Engineering, Faculty of Engineering, Kocaeli University, Kocaeli 41001, Turkey; kahramanpetek@gmail.com (P.N.K.); aaytac@kocaeli.edu.tr (A.A.); 2 Department of Polymer Science and Technology, Kocaeli University, Kocaeli 41001, Turkey; 3 Department of Medical Services and Techniques, Kocaeli Vocational School of Health Services, Kocaeli University, Kocaeli 41001, Turkey; huseyinuzuner@gmail.com; 4 Molecular Research and Antibody Laboratory, Department of Medical Microbiology, Faculty of Medicine, Kocaeli University, Kocaeli 41001, Turkey; 5 Department of Biomedical Engineering, Faculty of Engineering, Duzce University, Duzce 81620, Turkey; ikrimeucar@duzce.edu.tr

**Keywords:** double-layer nanofibers, electrospun, glutaraldehyde cross-linking, ciprofloxacin, purple carrot extract, drug release kinetics, Quasi-Fickian diffusion

## Abstract

To effectively manage bacterial infections and support tissue regeneration, developing multifunctional, biocompatible, and stable wound coverings is critical. Here, bilayer nanofiber wound dressings were developed via electrospinning, comprising a ciprofloxacin-loaded (CIP) polyvinyl alcohol (PVA) layer and a purple carrot extract-loaded (PCrt) polyvinylpyrrolidone (PVP) layer, and cross-linked with glutaraldehyde (GLA). Compared to the PVA/CIP-PVP control, which exhibited restricted swelling (~2.65%) and limited CIP release (8.51%), incorporating hydrophilic PCrt (7% *w*/*w*) enhanced macroscopic swelling to 707.14% and provided diffusion-controlled antioxidant capacity (12.86% via DPPH). Kinetic modeling identified Quasi-Fickian diffusion, while the Peppas–Sahlin model mathematically proved that severe chain entanglement and hydrogen bonding locked the matrix (negative relaxation constant), suppressing proportional release efficiency to ~22.8%. Biologically, the PCrt-free control showed GLA-induced basal cytotoxicity (66.31% viability) and 0% antibacterial activity due to strict drug confinement. However, PCrt addition restored L929 fibroblast viability to safe levels (75–77%) via antioxidant phenolic protection. Crucially, PCrt incorporation effectively mobilized residual GLA and phenolic compounds, elevating bactericidal efficacy to 100% against both *Escherichia coli* and *Staphylococcus aureus*. Although these bilayer nanofibers show strong synergistic bactericidal effects, crosslinking strategies on the green concept should be developed to eliminate GLA-induced toxicity and ensure wound management in safe clinical conditions.

## 1. Introduction

Applications that were interested in the wound healing process go back to 2500 BC [1]. This process is a complex physiological process that requires cell renewal and infection control [2]. Opportunistic pathogens such as *Escherichia coli* and *Staphylococcus aureus* cause bacterial infections, which delay the progressive wound healing phases such as hemostasis, inflammation, proliferation, and remodeling and prepare the way for chronic wound formation. Hydrogels, foams, transparent films, and those containing active substances, as well as nano/microfiber wound coverings, can be given as examples for studies that are being conducted on modern wound coverings [3,4]. Nanofibrous wound dressings fabricated by the electrospinning method stand out as promising systems since their high surface area-to-volume ratio, adjustable porosity, and structural properties similar to the extracellular matrix [5].

The configuration to be used can be selected according to the therapeutic function expected from the wound cover, such as single [6,7], coaxial [8,9], multilayer [10,11], and hollow fibers [12]. Since the wound healing process is complex, traditional single-layer wound coverings are often insufficient, especially for antibacterial protection and cellular proliferation. Biocompatible bilayer nanolayer wound coverings with their multifunctional properties serve as a compatible environment for wound healing thanks to their ability to allow the release of different bioactive agents [13,14,15].

Due to their flexibility during the electrospinning process, superior mechanical properties, and thermal stability, synthetic polymers are mostly preferred materials in wound dressing applications [5,16]. Polyvinyl alcohol (PVA) and polyvinyl pyrolidone (PVP), known for their biocompatible properties and cross-linking behavior with each other, are used together in electrogravity-based wound cover designs [17,18]. Wound exudate with a high liquid content causes wound coverings made of hydrophilic polymers to dissolve and lose their integrity. Various chemical cross-linking agents are used to overcome this problem and provide the physical strength and stability of the nanofiber structure in aqueous environments [19]. Glutaraldehyde (GLA) is a kind of cross-linking agent that stabilizes the nanofiber by forming a strong acetal network between polymer chains [19,20].

Controlled release properties of synthetic pharmacological agents and natural phyto extracts through the nanofiber give these structures a chance to activate tissue regeneration properties and to overcome the limited capacity of traditional polymeric scar coverings, which provide only passive physical support [21]. Also, a combination of synthetic antimicrobials with natural plant extracts can be used to develop multifunctional nanofiber designs [22,23]. Ciprofloxacin (CIP) is a kind of broad-spectrum fluoroquinolone antibiotic that has high efficacy in the control of infections [24]. This antibiotic exerts its effect on Type II topoisomerase enzymes—DNA gyrase and topoisomerase IV—which play a critical role in bacterial DNA replication. This effect leads to the suppression of DNA synthesis through complexes formed between the drug, enzyme, and DNA. Although these formed complexes are reversible in nature, they rapidly disrupt the bacterial growth process [25]. Ciprofloxacin is a frequently preferred antibacterial agent in nanolife-based systems. The porous structure of nanofibers allows for gas and moisture transfer, while the drug integrated into the nanofiber can be released in a controlled manner to maintain its antibacterial efficacy.

Purple carrot (PCrt) extract exhibits strong antioxidant and free radical scavenging properties at the in vitro level thanks to its phenolic compounds such as anthocyanins (especially ferulic acid derivatives) and chlorogenic acid [26]. Anthocyanins found in purple carrots are widely used as a natural dye source due to their strong coloring properties; they are considered a color additive in beverages, jellies, and confectionery products. Anthocyanins are flavonoid compounds found in fruits and vegetables, as well as in leaves, flowers, and grains. They perform functions such as providing photoprotection in plants, neutralizing free radicals, and attracting pollinators. These naturally glycosylated compounds can be bound with different aromatic and aliphatic acid derivatives, and it is reported that both the degree of glycosylation and acylation are decisive for bioavailability [26].

The release profile of bioactive components is regulated by the addition of natural plant extracts into polymeric nanofiber matrices. In addition, antioxidant, antimicrobial and regenerative properties of the extract significantly increase the therapeutic effectiveness of the wound cover [27,28]. Çetinkaya et al. integrated bioactive compounds derived from black carrots into nanofibrous structures containing PVA and tin dioxide with the help of the electrospinning method. The chemical stability tests show that the nanofiber coating protects the bioactive compounds against external factors and also ensures a significant increase in antioxidant capacity. In particular, the suppression of oxidative components demonstrates that the nanofibrous structures can effectively stabilize anthocyanins against degradation [29]. Another study conducted by Algarra and his team found that compared to traditional orange carrots, black carrot varieties have a higher polyphenol content and, accordingly, their free radical removal capacity significantly increases. It was also emphasized in the study that the acylated structure of anthocyanins plays a decisive role in both color stability and antioxidant activity [30].

Crucially, electrospun bilayer and multilayer nanofibrous scaffolds have emerged as advanced platforms capable of compartmentalized drug delivery and tailored release kinetics [31,32]. Recent studies on multilayer geometries demonstrate that incorporating conventional antibiotics into wound-facing contact layers yields an immediate, rapid burst release to establish a localized antibacterial barrier against opportunistic pathogens such as *Staphylococcus aureus* and *Escherichia coli* [31,32,33]. For instance, Soleiman-Dehkordi et al. (2024) developed a multilayer PVA/gelatin scaffold wherein amoxicillin in the wound-contact layer provided an early burst release (~49–59% at 24 h) to combat exogenous pathogens, while *Tanacetum polycephalum* essential oil in the middle layer underwent sustained, diffusion-controlled delivery to maintain a sterile zone and promote fibroblast migration [31]. Similarly, Güneş et al. (2027) engineered a double-layer PCL–PEO/PCL–chitosan membrane exhibiting rapid tigecycline release (98.8% within 120 min) alongside sustained quercetin diffusion (42% at 24 h), thereby modulating local drug exposure and enhancing cell viability [33]. Furthermore, layer-by-layer sandwich architectures—such as the PVA/gelatin/PVA membrane reported by Huang et al. (2023)—confirm that secondary polymer layers act as physical diffusion barriers, delaying inner bioactive release (e.g., curcumin) by over 55% compared to single-layer matrices [32].

Beyond release kinetic control, integrating natural bioactive components with synthetic antimicrobials provides essential multi-target therapeutic synergies [34,35]. While synthetic antibiotics (e.g., ciprofloxacin, levofloxacin, amoxicillin) rapidly suppress bacterial replication via DNA gyrase inhibition or cell wall disruption [31,35], natural phytogenic compounds—such as flavonoids, essential oils, and phenolic extracts—scavenge reactive oxygen species (ROS), attenuate prolonged inflammatory cascades, and enhance dermal fibroblast proliferation [31,34,35]. Monavari et al. (2024) demonstrated that co-axial core–shell nanofibers delivering levofloxacin alongside quercetin achieved synergistic therapeutic effects, significantly reducing bacterial load and oxidative stress to accelerate burn wound closure in vivo [35]. Additionally, Serinçay et al. (2013) showed that incorporating plant bioactives like Aloe vera into ciprofloxacin-loaded PVA/PAA mats stabilized the electrospun matrix, improved water/thermal stability, and enhanced anti-adherence efficacy against *S. aureus* and *P. aeruginosa* [34].

While the aforementioned studies often rely on the rapid burst release of synthetic antimicrobial agents—which can sometimes induce local cellular toxicity—our system introduces an alternative protective mechanism. Within the scope of this study, we aimed to develop a modern nanofiber-based wound dressing system capable of addressing the multifaceted nature of wound healing through controlled physical restriction and synergistic biochemical mobilization. Rather than reducing wound dressing design to the performance of a single material, this study focuses on a bilayer configuration. It differs from existing literature by establishing a functional division of labor between the layers; antibacterial protection is confined to the lower layer directly in contact with the wound bed and intentionally restricted by a chemical cross-linking network to prevent initial toxicity, while natural bioactive components (PCrt) that trigger tissue regeneration and mobilize the restricted matrix are positioned in the top layer.

For this purpose, an original double-layer nanofiber wound covering consisting of a Ciprofloxacin-loaded (CIP) PVA first layer and a purple carrot (PCrt) extract-loaded PVP second layer was developed to systematically investigate the physicochemical and biological effects of the formulation. Quantitative mathematical modeling (Peppas–Sahlin and Korsmeyer–Peppas kinetics) was employed to mathematically elucidate the severe physical confinement mechanism of the matrix. The effects of CIP and various PCrt ratios on the morphological structure, swelling behavior, and release kinetics were characterized in detail. Our principal findings demonstrated that while the drug-loaded control group exhibited high basal toxicity, the incorporation of PCrt successfully restored L929 fibroblast viability to biologically safe levels (75–77%) through antioxidant phenolic protection, while simultaneously achieving 100% bactericidal efficacy against both *E. coli* and *S. aureus* through synergistic phenolic release and controlled GLA mobilization.

## 2. Materials and Methods

### 2.1. Materials

Polyvinyl pyrolidone (PVP, mean molecular weight M_w_ ≈ 40,000 g/mol) and glutaraldehyde solution (50 wt% in aqueous solution) were obtained from Sigma-Aldrich (Steinheim, Germany). Polyvinyl alcohol (PVA, Kuraray Poval 49–88; with a viscosity value of 45.0–52.0 MPa.s, a hydrolysis degree of 86.5–89.0 mol% and an ash content of 0.4% by weight) was supplied by Arkem Kimya (Istanbul, Türkiye). Purple/black carrots (*Daucus carota* ssp. *sativus*) concentrate (liquid) (PCrt), used as a natural bioactive agent, was supplied by Gemma Food Colors (Istanbul, Türkiye). Ciprofloxacin (Cipro 500 mg, film-coated tablet), used as an antibacterial agent, was obtained from Biofarma İlaç Sanayi (Istanbul, Türkiye). The tablets were crushed and ground into a fine powder before being used in the preparation of the nanofibers. Hydrochloric acid (HCl, at a concentration of 37%) was purchased from Sigma-Aldrich (St. Louis, MO, USA). Acetone (analytical grade, ≥99.5%) was purchased from Sigma-Aldrich (Steinheim, Germany). Phosphate buffer solution (PBS) and 99.5% analytically pure ethanol were obtained from ChemBio Laboratory Research and Tekkim Kimya (Istanbul, Türkiye), respectively.

### 2.2. Methods

#### 2.2.1. Preparation of Solutions

Double-layered nanofiber matrices were prepared from PVA and PVP polymeric solutions. For the production of the first layer, PVA was dissolved in a pure water/ethanol (9.5:0.5, *v*/*v*) mixture at a rate of 7% (*w*/*v*). Homogenization of the prepared solution was performed by gradually heating the solution from 30 °C to 80 °C over a period of 3 h. After the addition of the CIP to the cooled solution at a rate of 5% by weight of the polymer mass, it was mixed at 30 °C for 24 h. For the second layer, PVP solution was prepared in a distilled water/ethanol (8:2, *v*/*v*) mixture at a concentration of 60% (*w*/*v*) and mixed at 60 °C for 3 h, then at 30 °C for 24 h.

#### 2.2.2. Double-Layer Nanofiber Production

Nanofibers were produced using an electrospinning device (Invenso, Basic System Electrospinning device, Nanospinner, Cambridge, MA, USA) by the application of the sequential electrospinning technique at 24 ± 2 °C and 65 ± 5% relative humidity. Process parameters were fixed at a 1.0 mL/h feeding rate, 19 kV voltage, and 15 cm collector distance for the production of all nanofiber groups. For the formation of the first layer, the PVA/CIP solution was applied to the aluminum collector for 1.5 h. In order to obtain double-layer nanofiber matrices, PVP/PCrt solutions were electrospun onto the first layer for 1.5 h under the same process parameters, containing empty, only CIP, and 3%, 5%, and 7% (*w*/*w*) PCrt added. Table 1 shows the composition and sample codes of the nanofibers produced by the electrospinning method.

#### 2.2.3. Chemical Crosslinking

**Figure 1:**
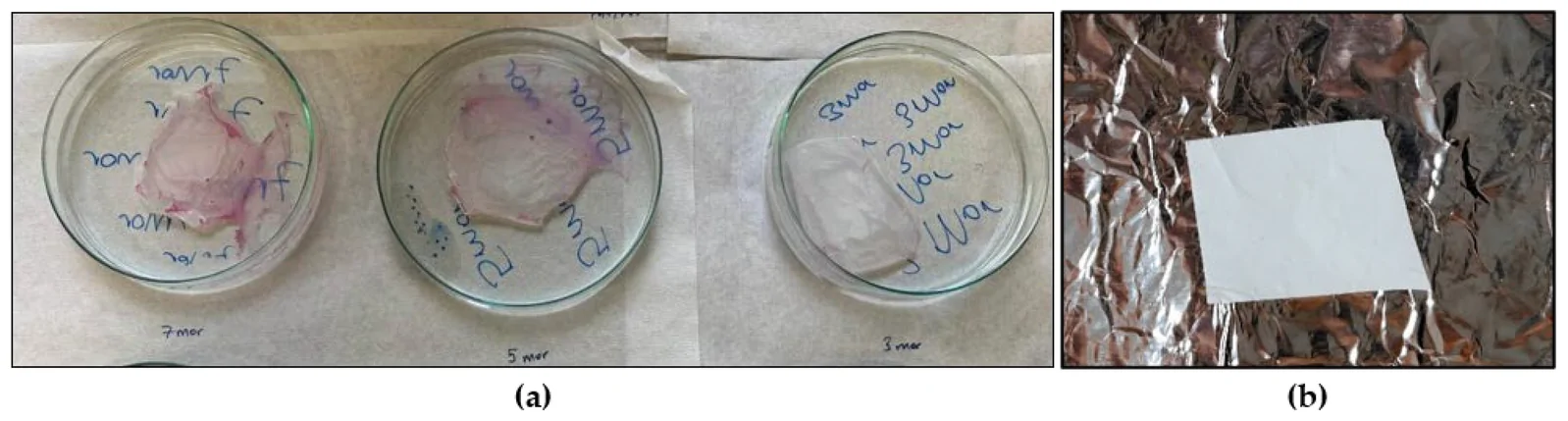
(**a**) Double-layer nanofiber matrices that have been cross-linked with glutaraldehyde solution; (**b**) the final wound cover that has completed the cross-linking process.

Finally, to increase the stability of nanofibers in aqueous media, nanofibers were cross-linked for 24 h at room temperature in a solution bath consisting of 0.15 M glutaraldehyde (50% *w*/*w*) in acetone (147.5 mL) and 0.05 M HCl (37% *w*/*w*) acting as a catalyst. Nanofiber membranes were washed with ethanol for 5 min and PBS three times, respectively, for the elimination of acidic residues. Then the samples were left to dry for 24 h at room temperature before characterization. Figure 1 shows the double-layer nanofiber matrices that have been cross-linked with glutaraldehyde solution, and the final wound cover that has completed the cross-linking process.

### 2.3. Characterization

Morphological surface analyses, diameter measurements of nanofibers, and structural integrity of nanofiber matrices were detected using a Thermo Scientific Quattro S model (Waltham, MA, USA) scanning electron microscope (SEM). With the help of high-resolution SEM images, numerical analysis of fiber diameter distributions was performed, and the diameter of 80 randomly selected fibers from each sample was measured using the ImageJ (Java 1.8.0_345, 64-bit, 1.54d Wayne Rasband and Contributors National Institutes of Health, USA) (National Institutes of Health, USA) analysis program. Origin Lab software (OriginPro 9.0, 64-bit, 1991–2013 OriginLab Corporation, Northampton, MA, USA) was used for the modeling of frequency distribution graphs and normal distribution (Gaussian fit) analyses of the raw data obtained from these measurements, and the average diameter and standard deviations of the fibers were determined.

To provide basic structural characterization of nanofibers, to detect the interactions between bioactive agents and to demonstrate the presence of the crosslinker on the polymeric structure, the Perkin Elmer Spectrum 100 brand ATR-FTIR device (Waltham, MA, USA) was used. The wavelength range of 650–4000 cm^−1^ was used for the spectroscopic 4 scans. Origin Lab software was used for processing the obtained raw spectra, examining specific band shifts, and for the comparative modeling of all sample groups on the same axis.

In order to evaluate the swelling capacity of the produced nanofiber structures, the samples were cut into 1 cm × 1 cm dimensions using a method reported in the literature [36]. Firstly, their dry weight (w_o_) was determined. Then each sample was placed in a medium containing 30 mL of distilled water and kept in distilled water for 24 h. At the end of this period, the free water on the surface of the fibers was removed quickly with the help of filter paper, and the final swollen masses (w_f_) of the samples were recorded. Finally, the swelling degrees of the layered nanofiber matrices were calculated using Equation (1).

(1) $$\mathrm{Swelling}\;\mathrm{capacity}\;(\%)=\frac{W_{f}-W_{o}}{W_{0}}\times 100$$

To examine the effect of different acidity and alkalinity levels on color sensitivity in nanofiber structures, in vitro pH stability tests were conducted. For this purpose, nanofiber samples were kept in buffer solutions prepared with pH values of 4, 6, 7, 8, and 10, respectively, for 10 days. The visual color differentiation developed by the active substances within the polymeric structure in response to pH variations was examined at the end of the ten-day contact period.

In addition to all these characterization tests, biological and bioactive property tests such as cell viability, antibacterial activity, in vitro drug release, and antioxidant assays were also conducted on the nanofibers.

#### 2.3.1. Cytotoxicity Test

WST-8/CCK-8 test protocol was applied based on direct contact of the samples with the L929 fibroblast cell line for the cytotoxicity analyses of the nanofibers [37]. The L929 mouse fibroblast cell line was obtained from the Kocaeli University Faculty of Medicine, Department of Microbiology (Kocaeli, Turkey) 30-min exposure to UV light was applied for the sterilization of the samples. Then, the nanofibers were transferred to 24-well plates and incubated at 37 °C in a 5% CO_2_ environment by seeding 7 × 10^4^ L929 cells on each sample surface. Following the 24-h incubation period, CCK-8 detection solution (Elabscience, Houston, TX, USA) prepared in a ratio of 1:10 in the medium was added to each well, and an additional 2-h incubation at 37 °C was performed. After this stage, 0.1 mL culture supernatant was transferred into the 96-well plates, and the colorimetric absorbance of each well was measured at 450 nm with the help of a microplate reader (Multiskan FC, Thermo Fisher Scientific, Waltham, MA, USA). In the study, cells without samples were evaluated as a negative cytotoxicity control, cells treated with 1% (*v*/*v*) Triton X-100 (Sigma-Aldrich, St. Louis, MO, USA) as a positive control, and 1:10 diluted CCK-8 solution prepared in culture medium in an empty well as a blank. All absorbance measurements were performed in triplicate, and the mean values were determined. Cell viability (%) was calculated according to Equation (2).

(2) $$\mathrm{Cell Viability}\left(\%\right)=\frac{A_{b,\;\mathrm{sample}}-A_{b,\;\mathrm{blank}}}{A_{b,\;\mathrm{negative control}}-A_{b,\;\mathrm{blank}}}\times 100$$

In Equation (2), A_b_ represents the absorbance of the sample nanofiber, A_b_ blank represents the absorbance of the empty well, and A_b_ negative control represents the absorbance of the negative control.

#### 2.3.2. Antibacterial Activity

The quantitative Agar Plate Colony Counting method, designed in accordance with the AATCC 100 test [37,38], was used to determine the antibacterial capacity of nanofibers. The standard bacterial strains of Gram-negative *E. coli* (ATCC 8739) and Gram-positive *S. aureus* (ATCC 6538P) were cultured on Nutrient Agar (Biolife Italiana, Milan, Italy) plates. Both sides of the nanofiber matrices were sterilized for 30 min with ultraviolet light and placed on 24-well culture plates aseptically. During the preparation of the bacterial inoculum, Nutrient Broth (NB) (Merck KGaA, Darmstadt, Germany) was used, diluted to a concentration of 1/20 with distilled water. Bacteria grown in Nutrient Agar (NA) medium were transferred to this dilute NB and brought to 0.5 McFarland standard turbidity level (600 nm absorbance value 0.08–0.1; 1 × 10^8^ CFU/mL) with the help of a spectrophotometer. Subsequently, the bacterial density was diluted to 1 × 10^5^ CFU/mL with 1/20 NB. One milliliter of this standardized solution was dropped onto the pre-sterilized nanofibers that were placed in the wells, and the samples were incubated for 24 h in a humid environment at 37 °C. At the end of the period, the liquid extracted from the bacterial solutions was subjected to neutralization in Soybean-Casein Digest Lecithin Polysorbate (SCDLP) medium. An aliquot (0.02 mL) of the neutralized liquid was taken and spread onto NA agar using a Drigalski spatula, and the plates were incubated for another 24 h at 37 °C. At the end of the process, the live colony counts on the plates were determined, and the percentage of the antibacterial activity was calculated according to Equation (3).

(3) $$\mathrm{Antibacterial}\;\mathrm{Activity}\;\left(\%\right)=\frac{B-A}{B}\times 100$$

Here, A and B represent the surviving cells (colony-forming unit/mL) at the end of the 24-h contact time on the plates containing test samples and controls, respectively. Three measurements were taken to average the results.

#### 2.3.3. In Vitro Drug Release

To obtain the standard calibration lines, CIP and PCrt stock solutions were prepared using PBS (phosphate-buffered saline) as the solvent, followed by applying the serial dilution technique to achieve the concentration ranges specified in Table 2. Standard solutions that were subjected to a 24-h incubation process at a temperature of 37 °C were used for the UV-Vis spectral analyses, which were performed in the 200–1000 nm range. The peak absorption wavelength (λ_max) of both active components (CIP and PCrt) was determined to be 290 nm according to the results of the conducted scans. The obtained spectrophotometric readings were transferred to the Origin program, and calibration graphs were plotted to form the basis for the quantitative analysis of the active ingredients. The concentrations of released CIP and PCrt were calculated using the standard calibration curves of the respective active agents. The linear regression coefficients (*R*^2^) for the calibration curves were highly reliable, being ≥0.997 for CIP and ≥0.996 for PCrt.

In vitro release profiles of nanofiber matrices were determined by using test samples with a mass of 20 mg that were placed in 5 mL of PBS and tested for 24 h in a shaking incubator at 37 °C. Liquid samples were collected at the 1, 2, 3, 4, 5, 6, and 24-h time points of the incubation period, and their absorbance (OD) values were measured at 290 nm. These recorded absorbance data were converted to concentration equivalents using standard calibration curves. To comprehensively evaluate the kinetic efficiency, the cumulative release percentage (%) of the formulations was calculated according to Equation (4).

(4) $$\mathrm{Cumulative}\;\mathrm{release}\;\left(\%\right)=\frac{V\;\sum_{\;i=1}^{\;n}C_{i}}{m}\times 100$$

Here, *m* represents the theoretical active agent mass loaded onto the nanofiber; *V* represents the PBS volume (5 mL); and *C_i_* represents the measured concentration at time point *i*. In order to evaluate both the total dosage capacity and the kinetic release efficiency of the matrices synergistically, the release profiles for all experimental groups (including PCrt-loaded matrices) were reported in terms of both absolute cumulative concentration and cumulative release percentage.

##### Mathematical Modeling of Release Kinetics

To elucidate the fundamental release mechanisms of the active agents from the cross-linked bilayer nanofiber matrices, the in vitro cumulative release data were fitted to five distinct empirical and semi-empirical mathematical models: Zero-order, First-order, Higuchi, Korsmeyer–Peppas, and Peppas–Sahlin. The kinetic modeling and non-linear regression analyses were performed using the DDSolver add-in program for Microsoft Excel.

To prevent the artificial inflation of the correlation coefficient caused by an increased number of model parameters, the adjusted coefficient of determination ($R_{\mathrm{adj}}^{2}$) was employed alongside the root mean square error (RMSE) as the primary statistical criteria to evaluate the goodness-of-fit. The model exhibiting the highest $R_{\mathrm{adj}}^{2}$ and the lowest RMSE was selected as the best-fitting model. Furthermore, the diffusional exponent (*n*) derived from the Korsmeyer–Peppas model was utilized to characterize the transport mechanism, classifying it as Quasi-Fickian diffusion (*n* < 0.45), Fickian diffusion (*n* = 0.45), non-Fickian/anomalous transport (0.45 < *n* < 0.89), or Case-II transport (*n* ≥ 0.89). The polymer relaxation constant (*k*_2_) from the Peppas–Sahlin equation was specifically analyzed to determine the structural state of the highly swollen matrix during the release process.

#### 2.3.4. Antioxidant Assays

The DPPH free radical scavenging test was conducted to evaluate the antioxidant potential of layered nanofibers following Ajmal et al. [39]. Layered nanofiber samples (25 mg) were mixed with 5 mL of 100 μM DPPH in ethanol and incubated for 30 min. under dark conditions at room temperature. At the end of the incubation, the absorbance values of the samples were measured at 517 nm by a UV-VIS spectrophotometer (Shimadzu Corporation, Kyoto, Japan). Equation (5) was used to calculate the antioxidant activities of the layered nanofibers.

(5) $$\mathrm{DPPH}\;\mathrm{Radical}\;\mathrm{Scavenging}\;\mathrm{Activity}\left(\%\right)=\frac{A_{\mathrm{control}}-A_{\mathrm{sample}}}{A_{\mathrm{control}}}\times 100$$

In this equation, A_control_ represents the initial absorbance value of the DPPH solution, while A_sample_ represents the absorbance value measured after incubation with the nanofiber sample. The data presented represent the averages obtained from three repeated experiments.

#### 2.3.5. Statistical Method

All experiments were performed in three independent replicates, and the results are presented as the mean ± standard deviation (SD). The SD values obtained from the repeated measurements were used to represent the variability of the experimental data and are presented as error bars in the corresponding graphs.

## 3. Results and Discussion

### 3.1. FTIR Results of Layered Nanofibers

**Figure 2:**
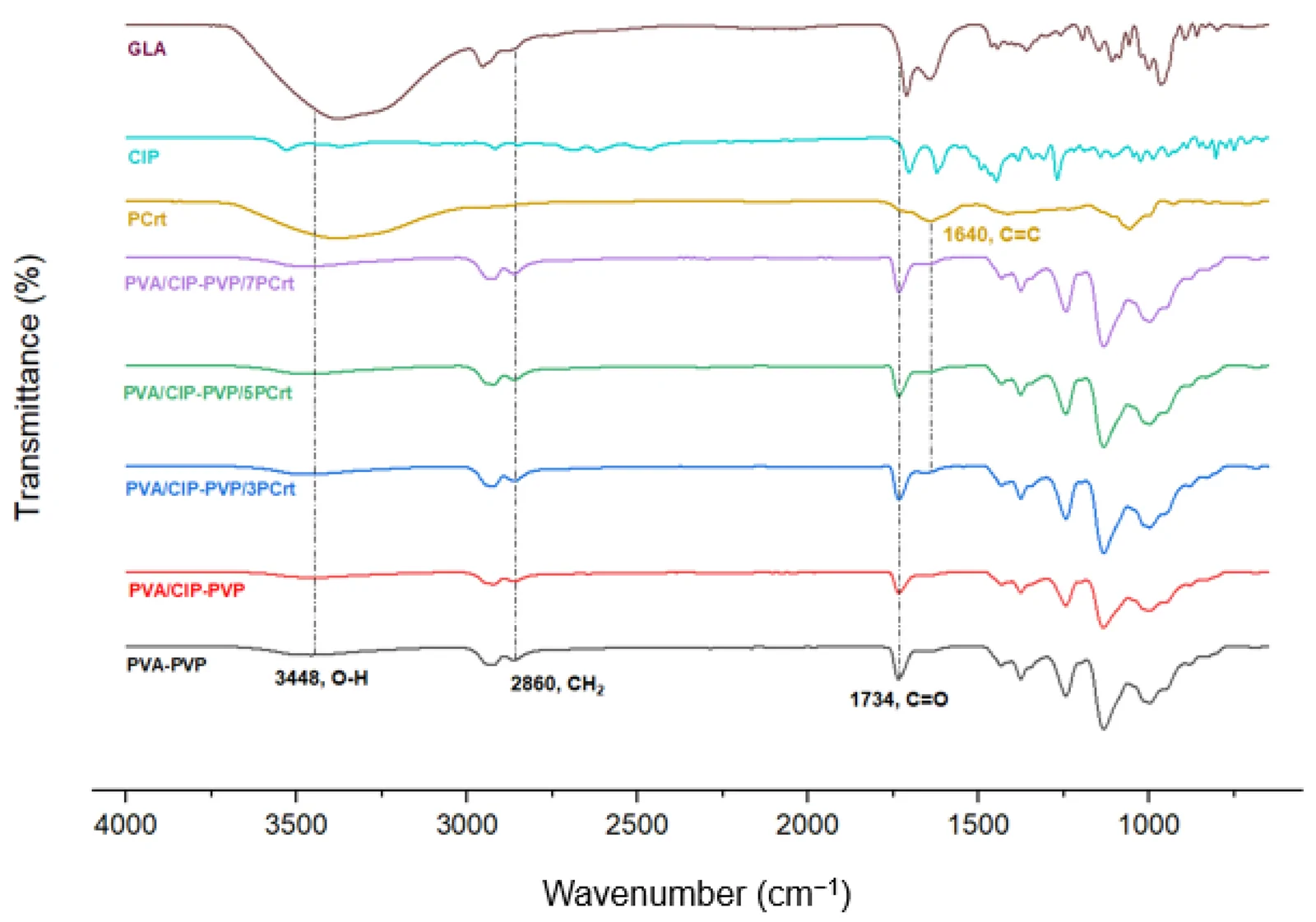
FTIR spectra of layered nanofibers.

FTIR analysis was applied to both surfaces of the layered nanofiber, and the resulting spectra were found to be identical. This result indicates that the nanofibers have a similar chemical structure on both surfaces. The functional groups and possible interactions among components in the chemical structure of layered nanofibers were investigated using FTIR spectroscopy, and the resulting spectra are shown in Figure 2.

As a result of the interactions between PVA and PVP nanofiber matrices, a partial overlap of the characteristic absorption bands in the spectrum was observed. The broad and intense peak observed in the 3200–3550 cm^−1^ range, as stated in the literature, originates from intramolecular and intermolecular hydrogen bonds formed between the hydroxyl (-OH) groups in the PVA chains and the carbonyl (C=O) groups in the pyrrolidone ring of PVP [40]. In this study, GLA, used to crosslink the PVA-PVP nanofiber, consumes the -OH groups around the 3448 cm^−1^ band to form acetal bonds. A decrease in peak intensity is observed in this case. When the cross-linking mechanism of GLA, a bifunctional molecule, within the polymeric nanofiber is examined, one of the aldehyde ends combines with the hydroxyl (-OH) groups on the PVA chains to form hemiacetal networks. However, due to steric effects and conformational constraints in the reaction medium, the second aldehyde end may not be incorporated into the polymer network. Thus, it can remain suspended in the medium [41,42,43]. Unreacted free aldehyde structures within the molecular polymeric nanofiber are observed at a wavelength of 2860 cm^−1^. It is also known in the literature that carbonyl (C=O) stretching of free aldehydes generally shows a strong peak in the 1720–1740 cm^−1^ region [42]. These free aldehydes, which could not be removed despite purification of the synthesized nanofibers by washing, persisted in the spectrum as a high-intensity carbonyl absorption band at 1734 cm^−1^ [43]. Although CIP is intrinsically an antibacterial agent [44], it is unlikely to form a covalent Schiff base with GLA due to the absence of a primary amine group in its structure and the steric and electronic constraints associated with the secondary amine in the piperazine ring [45,46]. However, this does not exclude the possibility of non-covalent interactions between CIP and unreacted GLA molecules within the polymeric network. The decrease in carbonyl peak intensity in the 1734 cm^−1^ band in the spectrum is thought to be a result of weak non-covalent interactions. Electrostatic attractive forces and hydrogen bonds occurring between CIP molecules and unreacted free aldehydes in the polymeric nanofiber can limit the molecular mobility of free carbonyl groups. This decrease in the accessibility of carbonyl bonds and the restricted vibrational freedom is interpreted as potentially leading to attenuation in the relevant FTIR signal. Similarly, the literature reports that non-covalent hydrogen bond interactions in polymer matrices lead to spectral changes by restricting the freedom of carbonyl stretching vibrations [47].

Studies on PCrt show that carrots exhibit different FTIR characteristic peaks depending on their main components (anthocyanins and pectin). The characteristic peaks of purple carrot pectin extracts are the stretching vibrations of hydroxyl (O–H) groups at 3385 cm^−1^. The peak at 1056 cm^−1^ corresponds to C-O-C glycosidic bonds and C-O stretching vibrations of structural carbohydrates (pectin, cellulose) within a complex plant nanofiber [48,49,50]. Compared to the PVA/CIP-PVP control group, the spectrum of PCrt-added nanofibers shows only a limited concentration in the hydroxyl (-OH) stretching band in the 3500–3200 cm^−1^ range, instead of the expected high increase in intensity. This situation is attributed to the fact that free GLA molecules in the nanofiber may interact with the phenolic -OH groups of anthocyanins through hydrogen bonding and other non-covalent interactions, while these interactions can reduce the availability of free hydroxyl groups and restrict the vibrational freedom of the hydrogen-bonded network formed by PCrt components with the polymeric nanofiber matrix. Furthermore, the proportional but limited growth observed in the peak at the 1640 cm^−1^ band depending on the increasing PCrt concentration is a result of the overlap of the characteristic aromatic C=C double bond stretching (1626–1662 cm^−1^) in the anthocyanin structure with the existing carbonyl absorption band [51,52].

### 3.2. SEM Micrographs of Layered Nanofibers

**Figure 3:**
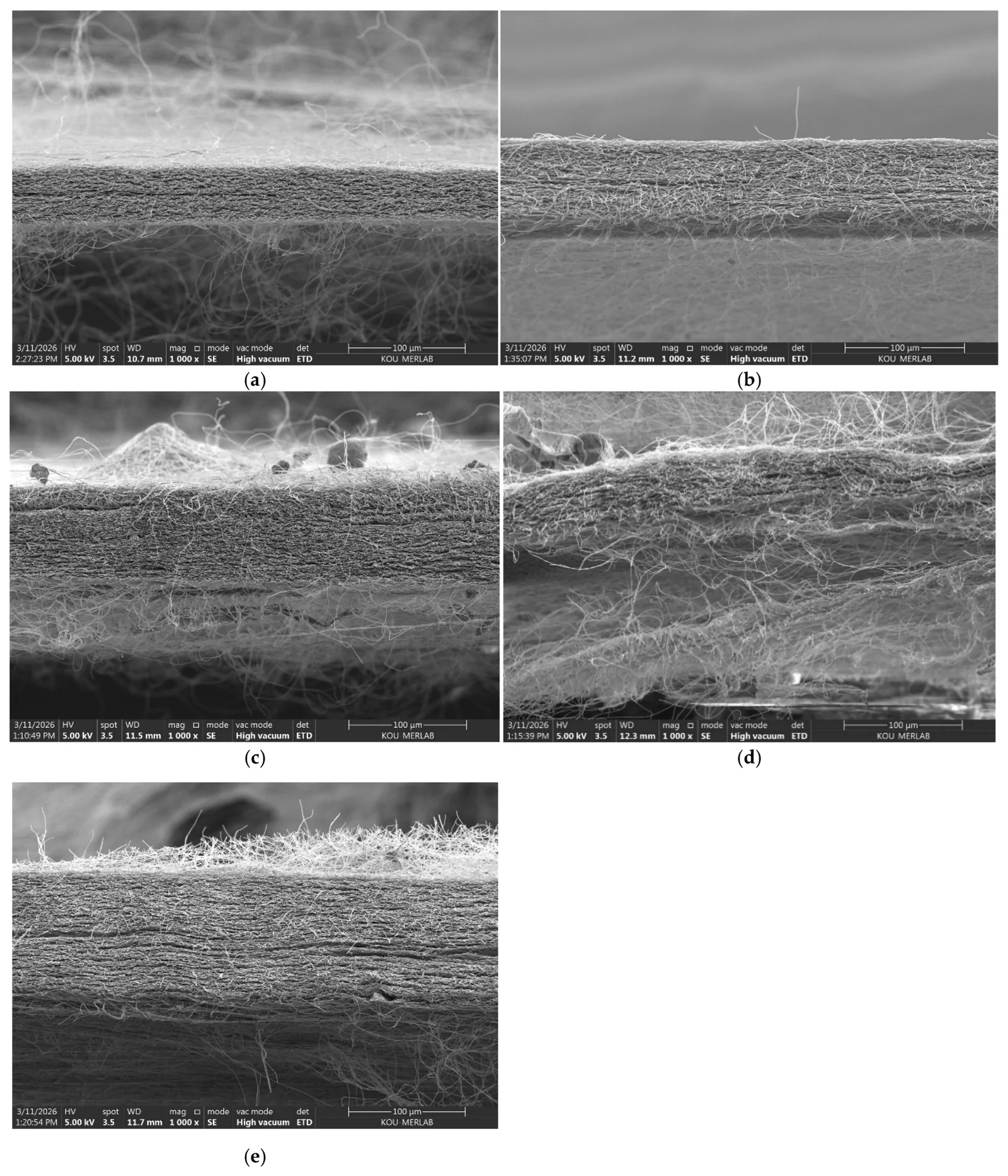
SEM micrographs at the 100 µm scale of the layered nanofibers: (**a**) PVA-PVP, (**b**) PVA/CIP-PVP, (**c**) PVA/CIP-PVP/3PCrt, (**d**) PVA/CIP-PVP/5PCrt, (**e**) PVA/CIP-PVP/7PCrt.

Cross-sectional SEM micrographs of the layered nanofibers are presented in Figure 3. The characteristic layered structure resulting from the sequential electrospinning process applied to create the layered structure is clearly observed in the SEM micrographs. Moreover, it was observed that all layered nanofibers were bead-free and in a regular fiber form. The narrowing of interlayer gaps and the compaction of the structure along the vertical axis in the PVA/CIP-PVP nanofiber compared to the PVA-PVP nanofiber demonstrate that CIP molecules bind the nanofiber with secondary bonds. When PCrt was included, anthocyanin aggregations were observed in the PVA/CIP-PVP/3PCrt and especially the PVA/CIP-PVP/7PCrt sections. This proves that the decrease in antioxidant activity observed at high concentrations is due to physical steric hindrance. In contrast, in the 5% PCrt formulation, a wavier and more reactive surface area morphology was observed, allowing for interlayer capillary diffusion. This structural optimization maximized performance by facilitating the release of the active ingredient.

**Figure 4:**
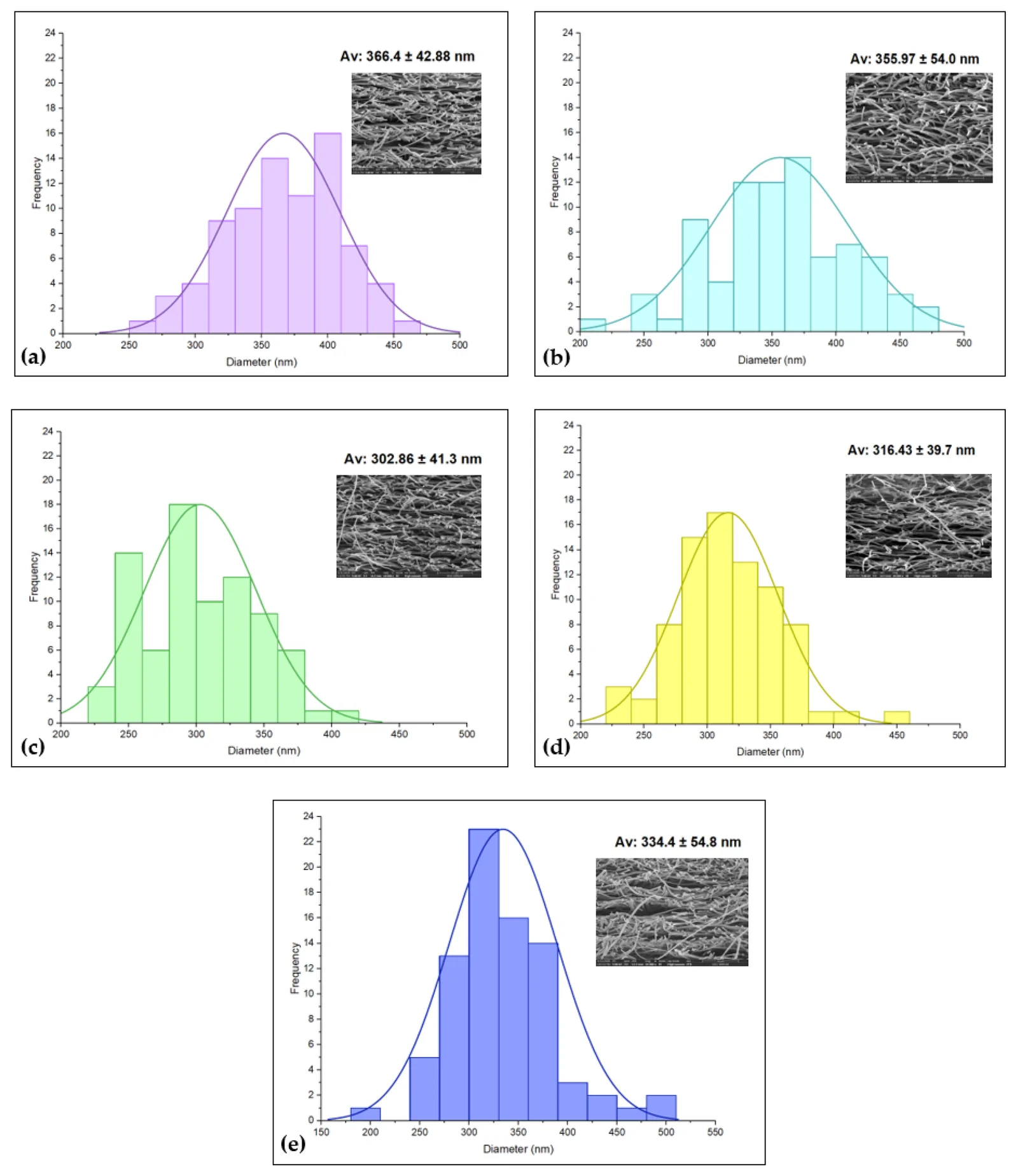
The average diameter distributions and SEM micrographs at the 10 µm scale of the layered nanofibers: (**a**) PVA-PVP, (**b**) PVA/CIP-PVP, (**c**) PVA/CIP-PVP/3PCrt, (**d**) PVA/CIP-PVP/5PCrt, (**e**) PVA/CIP-PVP/7PCrt.

The diameter distributions of the layered nanofibers and their SEM micrographs at the 10 µm scale are also presented in Figure 4, and a significant change in fiber diameters was observed depending on the nanofiber composition. When the diameter distributions of the nanofibers were examined at the 10 µm scale, a significant rheological and physicochemical trend was detected depending on the nanofiber content. The average diameter of the control group, PVA-PVP nanofiber, was measured as 366.4 ± 42.88 nm, and this value decreased to 355.97 ± 54.0 nm when CIP was included in the system. This limited diameter reduction indicates that CIP partially increases the electrical conductivity of the polymer solution, supporting the jet stretching forces under the electric field [53,54].

Concentration-dependent structural changes in nanofiber diameters were observed with the inclusion of PCrt extract in the system. When 3% PCrt was added to the formulation (PVA/CIP-PVP/3PCrt), the fiber diameter decreased to 302.86 ± 41.3 nm, resulting in the finest morphology. This sharp decrease is attributed to the fact that the anthocyanins, natural salts, and organic acids present in the PCrt extract increase the net charge density and electrical conductivity of the polymer solution [51]. The increased conductivity maximized the repulsive forces in the Taylor cone, causing the polymer jet to stretch and thin much more under the electric field [53,54].

However, when the PCrt concentration was increased to 5% and 7%, the nanofiber diameters increased to 316.43 ± 39.7 nm and 334.4 ± 54.8 nm, respectively, exhibiting a regular thickening profile. The addition of high concentrations of extract led to an increase in viscosity due to the molecular aggregation of dissolved active components (anthocyanins, organic acids, and polyphenols) within the nanofiber [29,51,52]. Chain entanglement and flow resistance resulting from viscosity acted dominantly over electrical tensile forces, preventing further thinning of the polymer jet and consequently leading to the production of thicker fibers [55,56,57].

### 3.3. Swelling Capacity of Layered Nanofibers

The low swelling capacity (2.80%) observed in PVA-PVP layered nanofibers is attributed to the dense acetal cross-linking network formed by GLA molecules [42]. When CIP was loaded into the nanofiber, this rate decreased to 2.65%. The active functional groups (carboxyl, amino, and carbonyl groups) in the CIP structure form hydrogen bonds with the hydroxyl and carbonyl ends in the polymer, hindering the penetration of water molecules into the polymer networks and reducing the swelling degree [58]. Furthermore, the rigid character of CIP molecules incorporated into the PVA structure restricts the mobility of the polymer chains and reduces the free volume through which water molecules can diffuse [59]. Consequently, due to these molecular constraints, a limited decrease in the water-holding capacity of CIP-loaded nanofibers occurs compared to control groups [60].

**Figure 5:**
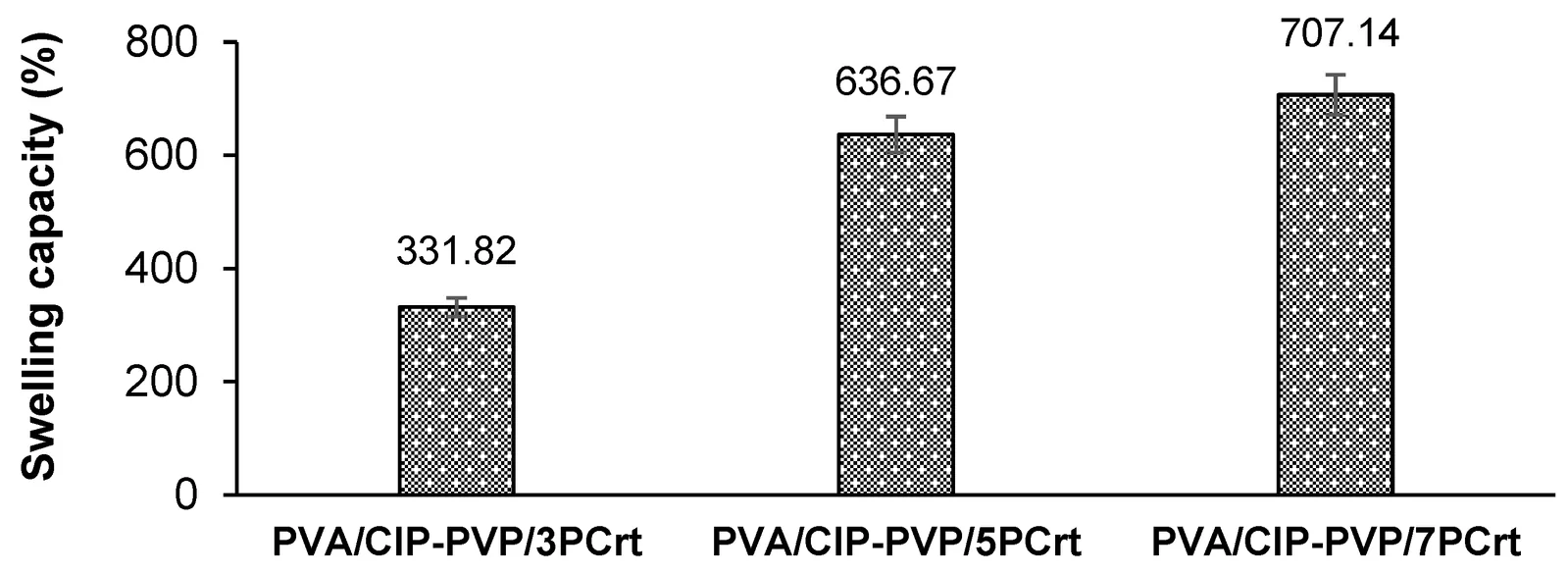
The swelling capacity of the layered nanofibers.

The results regarding the swelling ratios of the nanofibers and their concentration-dependent changes are presented graphically in Figure 5. The swelling ratio, which was 331.82% in the nanofiber containing 3% PCrt, increased to 636.67% with the addition of 5% PCrt and reached its highest level of 707.14% at a 7% PCrt concentration.

Chemically, the rich phenolic hydroxyl (-OH) groups in the structure of PCrt anthocyanins dramatically increased the overall hydrophilic character of the layered nanofibers [52], driving massive water uptake into the matrix. However, as corroborated by the restricted in vitro release profiles and the highly negative polymer relaxation constant (*k*_2_) in the Peppas–Sahlin kinetic model, this high macroscopic swelling does not translate to the unrestricted liberation of the bioactive agents. Although the hydrophilic groups draw pure water into the structure and expand the macroscopic volume up to 700%, the active molecules remain physically anchored to the swollen polymer network via dense non-covalent hydrogen bonding and severe chain entanglement (as observed in SEM aggregations). Consequently, the system acts as a highly swollen but diffusion-restricted nanofibrous matrix, where immense water affinity coexists with strict physical entrapment of the loaded agents.

### 3.4. pH Sensitivity Results of Layered Nanofibers

Cyanidin derivatives and acylated anthocyanins found in purple carrot extract undergo structural isomerization depending on the pH level of the environment, resulting in their halochromic properties [61,62,63]. In situ monitoring of pH changes in the wound microenvironment provides extremely critical clinical data about healing physiology and stages. While healthy epidermal tissue has a slightly acidic profile (classically pH 5.4–5.9), the acute wound environment approaches neutral/slightly alkaline (pH ≈ 7.4) levels with the disruption of epidermal integrity, and can shift to the alkaline spectrum (pH 7.5–9.0) due to bacterial activity and metabolites in case of chronic or infected wounds [64].

**Figure 6:**
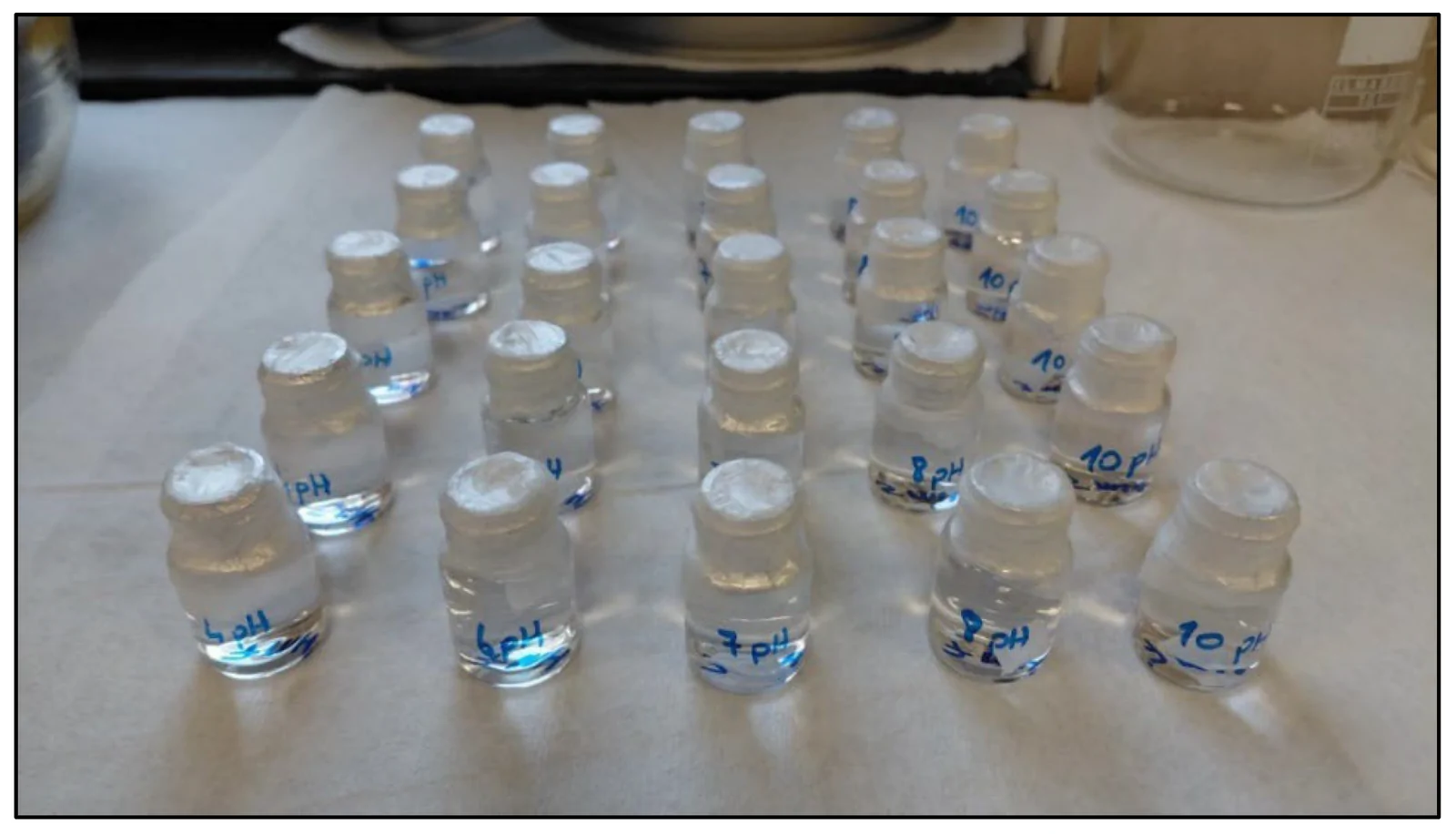
Appearance of layered nanofibers after pH sensitivity test.

In pH sensitivity tests of layered nanofibers, both the nanofibers and the test liquids remained completely colorless in different pH buffer solutions (pH 4, 6, 7, 8, and 10). The appearance of the layered nanofibers after the pH sensitivity test is given in Figure 6. A distinct pink coloration was observed in the fiber structure upon immersion in a GLA cross-linking bath. This phenomenon was explained by the conversion of anthocyanin pigments into the stable pink/red color-producing flavylium cation form as a result of the low pH conditions in the acid-catalyzed cross-linking bath [62,65,66]. The literature mentions that the delocalized electronic structure of anthocyanins undergoes a change in acidic environments through protonation reactions to form a stable flavylium cation tautomer, and this form produces red/pink color tones in the nanofiber [63,66]. However, the washing step after cross-linking led to the leaching of highly water-soluble anthocyanin pigments into the rinsing water. The complete disappearance of the observed pH-sensitive color transition can be explained by a diffusion mechanism associated with swelling and weak non-covalent interactions [67,68]. Purple carrot anthocyanins are glycosylated, hydrophilic, and water-soluble phenolic pigments [69,70].

Within the electrospun bilayer matrix, PCrt anthocyanins are expected to be retained within the PVP-containing top layer predominantly through physical entrapment and non-covalent interactions, including hydrogen bonding, rather than through specifically designed covalent conjugation [71,72,73]. Similar hydrogen-bond-mediated anthocyanin immobilization has been reported in electrospun polymeric nanofiber matrices [72]. In contrast, GLA crosslinking primarily stabilizes the PVA-containing bottom layer through the formation of acetal linkages between hydroxyl groups of PVA and the aldehyde groups of GLA [41,74]. Therefore, the applied GLA crosslinking strategy was not specifically designed to covalently immobilize the anthocyanin fraction within the PVP-containing layer.

The penetration of water into the hydrophilic matrix during contact with the aqueous rinsing bath leads to rapid swelling, which can facilitate the dissolution and diffusion of anthocyanins physically retained in the matrix into the aqueous phase [75,76]. In the literature, it has been reported that high water intake and swelling accelerate the release of anthocyanins; in addition, polyphenolic compounds can interact with hydrophilic polymers such as PVP through hydrogen bonds [72,77]. Accordingly, the high swelling degree of 707.14% observed in the current system may have contributed to the weakening of the retention capacity of anthocyanins in the matrix during the washing step and, accordingly, the loss of pH-sensitive halochromic color response.

To address this limitation and suppress anthocyanin leaching while stabilizing the pH-responsive performance for future translational applications, several structural modification strategies can be explored.

Chemical anchoring of anthocyanins onto polymeric backbones—such as covalent grafting onto dialdehyde cellulose nanofibrils via nucleophilic addition [68] or onto chitin matrices through in situ Mannich reactions [78]—provides a robust barrier against water-induced leaching, retaining full halochromic sensitivity even under humid or washing conditions.

Tuning matrix swelling by incorporating non-toxic green cross-linkers like citric acid [79] or constructing dynamic Schiff-base networks (e.g., oxidized sodium alginate with carboxymethyl chitosan) [73] effectively increases the gel content, constrains excessive water absorption, and locks chromophores within the polymer matrix.

Entrapping anthocyanins within biopolyelectrolyte nanocomplexes [80] or porous metal–organic frameworks (such as Zn-BTC MOFs) [81] physically shields the chromophores from rapid solvent dissolution while maintaining colorimetric responsiveness to target volatile gases [81,82].

Integrating these covalent coupling, green cross-linking, or nano-encapsulation approaches into the bilayer electrospinning framework represents a promising perspective to overcome wash-out instability and ensure long-term, reliable pH sensing in moist clinical wound environments.

### 3.5. In Vitro Drug Release of Layered Nanofibers

**Figure 7:**
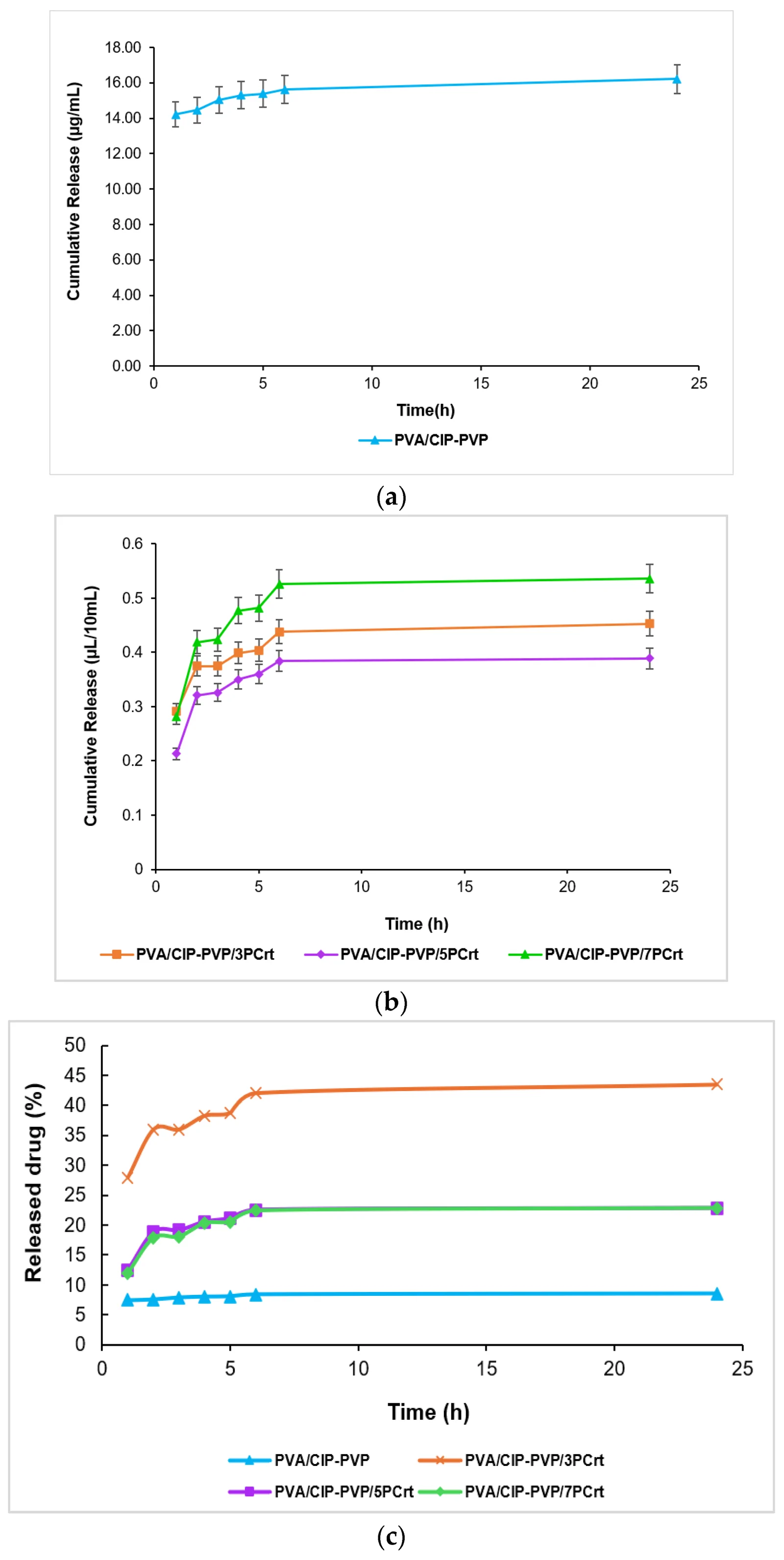
Time-dependent *in vitro* release profiles of the cross-linked bilayer nanofiber matrices: (**a**) Absolute cumulative release concentration (µg/mL) of CIP from the control PVA/CIP-PVP matrix; (**b**) absolute cumulative release amount (µL/10 mL) of the extract from PCrt-loaded formulations; and (**c**) cumulative percentage release efficiency (%) of all experimental groups.

In order to comprehensively understand the dosage capacity and basic transport mechanisms, time-dependent in vitro release profiles of layered nanofibers were evaluated both in terms of absolute amount released (Figure 7a,b) and cumulative percentage release (Figure 7c), showing a marginal increase from 14.2 µg/mL at 1 h to 16.2 µg/mL at 24 h. For PCR-loaded groups (Figure 7b), the absolute amount of the extract released into the medium reached its highest level (0.536 µL/10 mL) in the 7% PCrt formulation. This shows that the wound cover is able to provide higher absolute doses of bioactive phenolic compounds to the local microenvironment as the initial loading concentration increases.

However, when these absolute quantities are normalized to the cumulative percentage emission to evaluate the kinetic efficiency of the matrix (Figure 7c), a highly nonlinear, suppressive matrix effect occurs. The PVA/CIP-PVP group exhibited a severely restricted maximum proportional release of only 8.51% over a 24-h period. Additionally, while the PVA/CIP-PVP/3PCrt formulation exhibited 43.5% release, increasing the loading of PCrt to 5% and 7% markedly restricted the release efficiency by exhibiting a plateau of approximately 22.8%. This dilemma in lower proportional delivery forms despite higher absolute release, contraction of the free volume in matrices due to high loading, increased barrier effects, and dense molecules, is attributed to these factors [83,84]. As confirmed by SEM findings, high PCrt concentrations increased fiber diameters and led to chain entanglement. It is known that phenolic hydroxyl (-OH) groups, which are abundant in purple carrot extract, form a dense physical crosslink network through hydrogen bonds with PVA and PVP chains [52,85,86]. It is thought that this physical entanglement, which exhibits a synergistic effect with the chemical GLA cross-links, may have created a steric hindrance. It has a high macroscopic swelling capacity (e.g., 707.14%), however, it has been interpreted by us that the active agents remain firmly imprisoned in the entangled amorphous network.

Experimental release data were applied to five basic mathematical models, namely zero-degree, first-degree, Higuchi, Korsmeyer–Peppas, and Peppas–Sahlin, to explain the simultaneous in vitro release behavior of active compounds from the bilayer nanofiber matrix (Table 3). The success of the fit of the mathematical models was evaluated based on the adjusted coefficient of determination ($R_{\mathrm{Adj}}^{2}$) and root mean square error (RMSE) parameters. The use of the $R_{\mathrm{Adj}}^{2}$ value instead of the standard *R*^2^ is accepted as the basic condition in the literature to prevent the artificial increase in compliance caused by the increase in the number of parameters in complex polymeric matrix systems and to choose the most accurate model [87]. When the experimental data were examined, it was seen that classical linear models such as zero-degree, first-degree and Higuchi exhibited negative $R_{\mathrm{Adj}}^{2}$ values (−274.134 to −3.249) and high RMSE errors in all formulations. This statistical mismatch proves that the release of active substances from the two-layer nanolip matrix cannot be explained by simple diffusion or superficial matrix erosion mechanisms alone [88,89,90]. In contrast, semi-empirical and nonlinear models showed an extremely high correlation with the data. In particular, the Peppas–Sahlin model provided the highest correlation coefficients ($R_{\mathrm{Adj}}^{2}$ = 0.895–0.929) and the lowest residual errors (RMSE = 0.124–1.480) in all samples, giving a significant superiority to the standard Korsmeyer–Peppas model ($R_{\mathrm{Adj}}^{2}$ = 0.596–0.853). This superiority of the Peppas–Sahlin model is due to its ability to describe the processes of Fickian diffusion (*k*_1_) and polymer chain relaxation (*k*_2_) that occur during polymeric matrix hydration as two simultaneous and independent components [91,92,93]. The calculated n values for all formulations are between 0.044 and 0.150 and are considerably below the theoretically lower bound of 0.45 [89,92]. In the literature on polymeric nanofibers and microparticles, the case *n* < 0.45 is defined as “Quasi-Fickian” diffusion [94,95]. In electroplated nanofiber networks, this situation is explained by the rapid dissolution of the active molecules in the outer layer at first due to the high specific surface area of the fibers, followed by the slow diffusion process through the curved inner pore paths [94,95,96,97].

When the Peppas–Sahlin model parameters were examined, it was found that the Fickian diffusion constants were positive (*k*_1_ = 10.773–36.369), and the polymer relaxation constants were negative (*k*_2_ = −2.352 ile −7.454). A negative k_2_ value in the drug release kinetics literature, especially in PVA and hydrophilic polymer-based fiber/film systems, indicates that the relaxation and rearrangement of polymer chains have a restrictive/retarding effect instead of accelerating the release [98,99]. Specifically, the negative k_2_ constant calculated in this study mathematically confirms that the above-mentioned GLA covalent bonds and dense hydrogen bonds lock the matrix at a microscopic level and cause free volume contraction.

Finally, the addition of purple carrot extract to the PVP layer induced a concentration-dependent bidirectional change in the release rate constants (k). Adding PCrt to the matrix at 3% significantly increased the release rate constants compared to the unadulterated PVA/CIP-PVP matrix, the k_KP_ value increased from 7.510 to 31.827, and the k_1_ value increased from 10.773 to 36.369. It is thought that the highly hydrophilic structure of anthocyanins and phenolic compounds, which are abundant in purple carrot extract, may have increased the wettability of the matrix by reducing the contact angle with water, and this accelerated the release of active substances from the high-solubility PVP layer in the first place. However, when the PCrt ratio increased to 5% and 7%, a significant decrease in the release rate constants was observed, k_KP_ decreased to 16.153 and 15.406, respectively, while k_1_ decreased to 16.378 and 15.207, respectively. This sharp decrease observed in mathematical models directly coincides with the increasing physical chain entanglement with increasing concentration and blocking the diffusion pathways of phenolic aggregates.

It should be noted as a methodological limitation that both CIP and PCrt exhibit peak optical absorption at 290 nm. Consequently, in the dual-loaded nanofiber groups, the UV-Vis spectrophotometric analysis captures a combined absorbance signal, representing the net cumulative release of both CIP and PCrt, rather than allowing for their independent quantification. The baseline restricted release of CIP was independently established using the PCrt-free PVA/CIP-PVP control group. The substantial concentration increases observed in the PCrt-loaded formulations reflect the combined synergistic release resulting from the mobilized matrix. To achieve precise independent quantification of each component without spectral interference, advanced chromatographic techniques such as HPLC should be employed in future studies.

### 3.6. Antibacterial Activity of Layered Nanofibers

**Figure 8:**
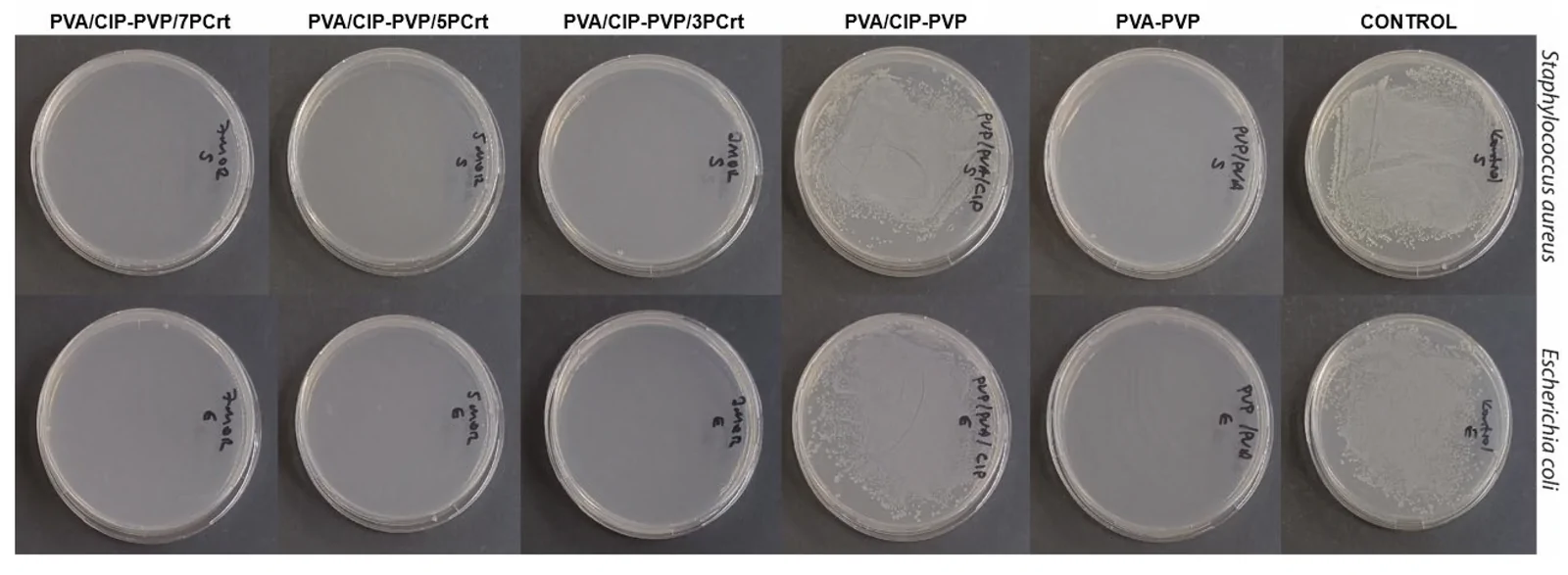
Agar plate images of layered nanofibers against *E. coli* and *S. aureus*.

**Figure 9:**
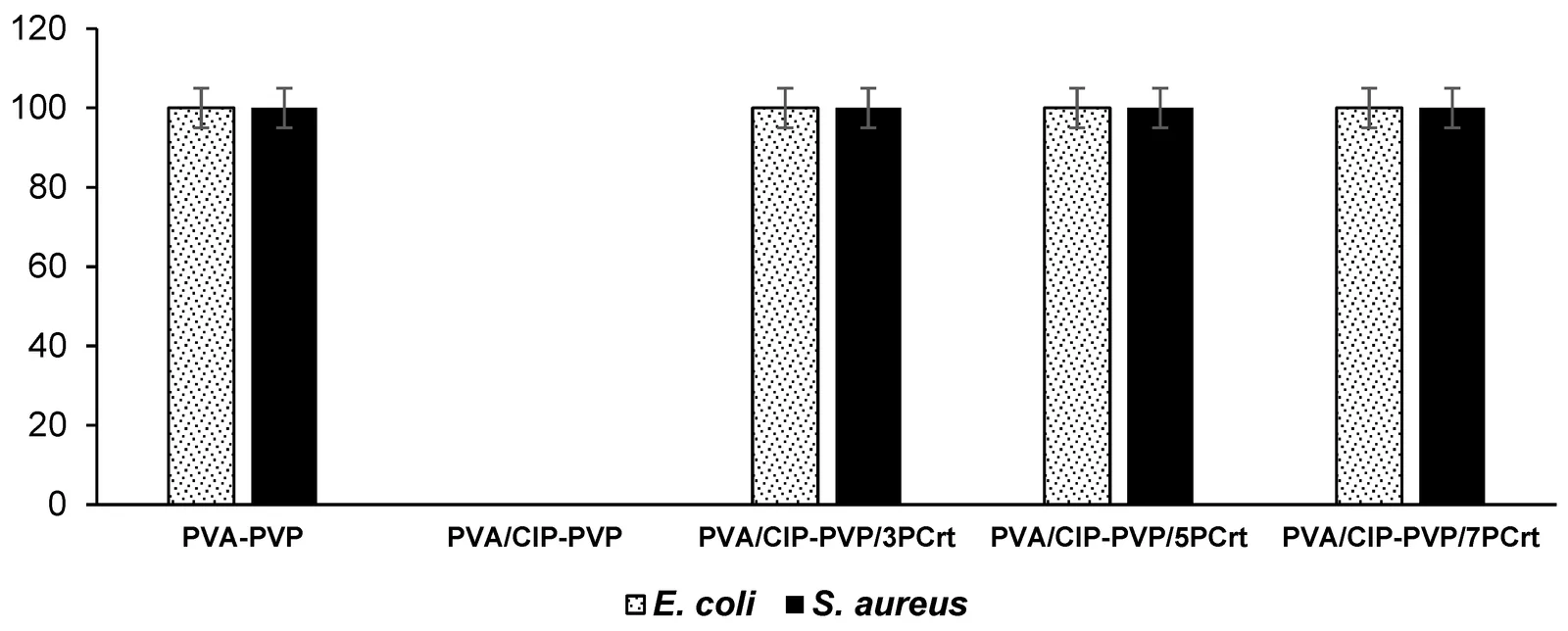
Antibacterial activity of layered nanofibers against *E. coli* and *S. aureus*.

Agar plate images and graphs of the results of the antibacterial activity of layered nanofibers against *E. coli* and *S. aureus* bacteria are shared in Figure 8 and Figure 9, respectively.

The observed reduction in bacterial count for the unloaded PVA-PVP matrix cross-linked with GLA is attributed to surface-associated residual acidic cross-linking conditions rather than intended antimicrobial activity. GLA is employed strictly as a structural cross-linking agent to impart aqueous stability to hydrophilic PVA/PVP chains, and does not serve as a primary therapeutic antibacterial agent in the dressing design.

According to the literature, PVA and PVP are biocompatible and hydrophilic polymers that do not possess antibacterial activity [37,100,101].

While full antibacterial activity was observed in PVA/PVP nanofibers cross-linked with GLA, no significant antibacterial activity was detected in layered PVA/CIP-PVP nanofibers containing CIP. This difference suggests that GLA alone does not suppress antibacterial activity; rather, the bioavailability of CIP may be limited in the CIP-containing system. Because CIP, as an active ingredient, has been proven to be a potent and broad-spectrum antibacterial agent effective against both Gram-negative and Gram-positive bacteria [44], the absence of detectable antibacterial activity in PVA/CIP-PVP layered nanofibers cannot be attributed to a lack of intrinsic antibacterial activity in CIP.

Given that CIP itself possesses strong antibacterial activity, the loss of activity in the PVA/CIP-PVP system is considered to be related to the immobilization of CIP within the cross-linked polymeric network rather than the disappearance of its intrinsic antibacterial property. Indeed, in the release study, the total CIP release was determined to be only 8.51% after 24 h, and the amount of CIP released increased from 14.2 µg/mL at 1 h to only 16.2 µg/mL at 24 h. This low release indicates that the majority of CIP is retained within the nanofiber matrix, and the amount of free CIP that can reach the bacterial environment remains limited. Possible hydrogen bonds and other non-covalent interactions between unreacted GLA groups and CIP, as suggested in the FTIR results, along with CIP–PVA interactions and the GLA-derived cross-linked network structure, may have further restricted the molecular mobility and diffusion of CIP. Therefore, the lack of observed antibacterial activity in PVA/CIP-PVP layered nanofibers can be explained by the limited release of CIP within the cross-linked nanofiber network and its consequent low bioavailability, rather than by GLA suppressing antibacterial activity or CIP losing its intrinsic activity.

However, it was found that adding different ratios of PCrt extract to the nanofibers resulted in 100% antibacterial inhibition in *S. aureus* and *E. coli* strains. In the literature, it is known that the rich cyanidin derivatives and phenolic acids in purple carrot extract exhibit strong inhibition of pathogenic bacteria [102] and suppress the growth of microorganisms such as *E. coli* [103]. Phenolic compounds obtained from carrot byproducts have been reported to show direct dose-dependent antimicrobial activity against both *S. aureus* and *E. coli* strains [104]. This high antibacterial effect is explained by the fact that anthocyanins, thanks to their delocalized electronic structures, disrupt bacterial cell membrane permeability, lead to the leakage of potassium (K^+^) ions out of the cell, and exhibit bactericidal or bacteriostatic activity by inhibiting protein synthesis [105]. Although the kinetic modeling demonstrated that the proportional release of PCrt was severely restricted (approx. 22%) at high loading concentrations due to matrix entanglement, these results indicate that the absolute concentration of the released phenolic compounds is still sufficient to vastly exceed the Minimum Inhibitory Concentration (MIC) required for *S. aureus* and *E. coli*.

The results suggested that, in addition to the performance of the bioactive components, the hydrophilic character of PCrt, stemming from its phenolic structure, also played a crucial role in drug release. Literature indicates that increased surface wettability, resulting from the higher hydrophilic anthocyanin content in the matrix, facilitates the diffusion of solvent molecules and ions into the polymeric network [106].

This structural alteration was considered to promote the swelling and mobilization of the densely cross-linked matrix, thereby facilitating the diffusion and release of natural active phenolics (PCrt) and previously constrained CIP molecules from the swollen matrix to achieve complete pathogen eradication.

As a study limitation, quantitative chromatographic techniques (such as HPLC or GC-MS) were not utilized in the present work to measure the precise concentration of unreacted free GLA residues. Instead, the biological safety threshold of leachable cross-linker species was established directly through in vitro cytotoxicity testing on L929 fibroblasts according to ISO 10993-5 criteria, where PCrt-containing matrices maintained cell viability at biologically safe levels (75–77%). Furthermore, un-crosslinked PVA/PVP control mats could not be evaluated in 24-h aqueous antibacterial assays due to immediate polymer dissolution upon liquid contact. Nevertheless, comparing the PCrt-free PVA/CIP-PVP control (0% antibacterial activity) with PCrt-loaded formulations (100% bactericidal efficacy) confirms that residual GLA alone does not drive complete pathogen clearance. Future translational studies will focus on quantifying residual cross-linkers via derivatized HPLC analysis and adopting green or physical cross-linking strategies to eliminate synthetic cross-linker toxicity. Although the material has not been found cytotoxic in PVA/alginate nanofibers crosslinked with GLA in the literature, it has been reported that GLA has a slight negative effect on cell viability, as in our study [107]. However, cell viability was increased to an acceptable level with the addition of PCrt in our study, and in a therapeutic nanofiber dressing designed as in our study, especially when a special bioactive or antimicrobial agent is included in the system, the antimicrobial contribution of GLA should be evaluated as a complementary effect, not as the main treatment mechanism.

### 3.7. Cytotoxicity Test Results

**Figure 10:**
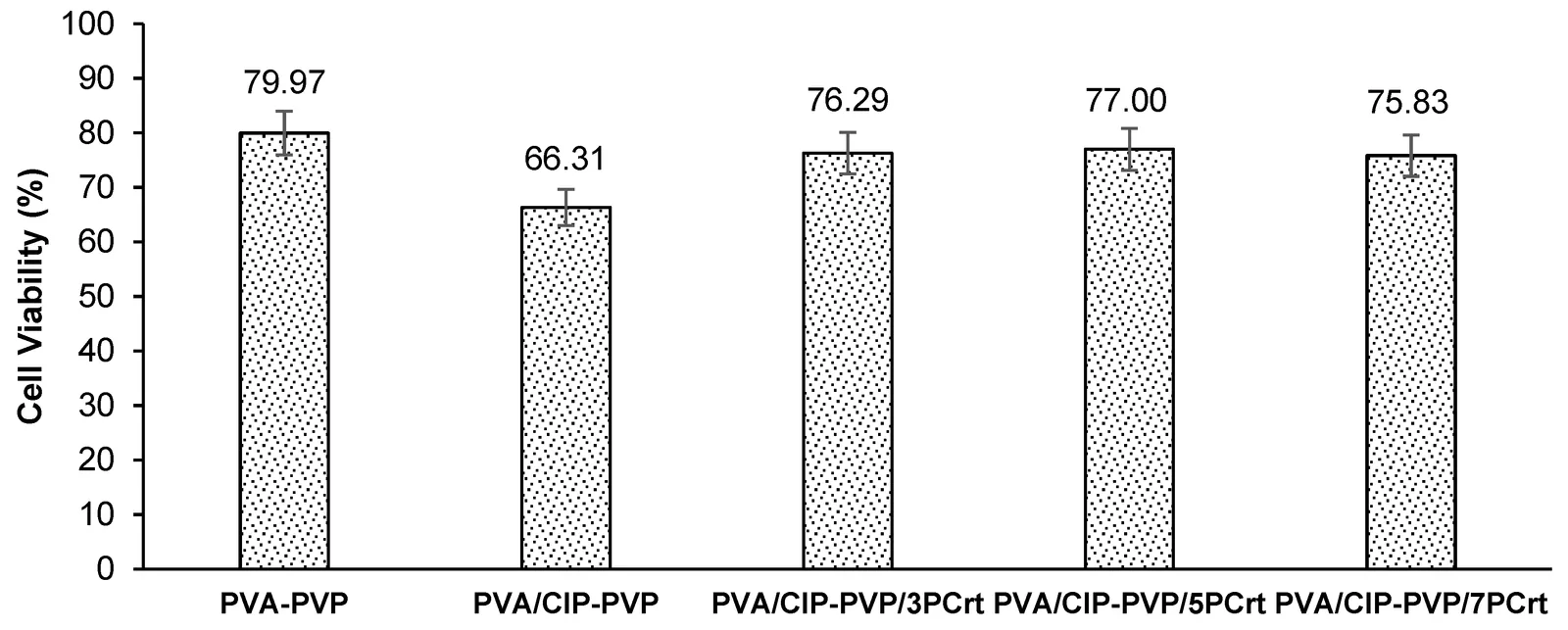
The cell viability test results for layered nanofibers.

Figure 10 shows the cell viability test results for layered nanofibers. During the test, the cell viability of the control was found to be 100%. The cell viability of the PVA-PVP nanofiber was determined to be 79.97%. It is thought that the failure to reach 100% cell viability in this layered nanofiber structure, composed of PVA and PVP polymers with inherently high biocompatibility, is due to the GLA cross-linking process applied to increase aqueous stability. Free aldehyde groups that remain in the network structure despite the washing process after cross-linking can interact with cells and cause mild cytotoxic stress [108,109,110]. Furthermore, it has been reported that the high electric field applied during the electrospinning process causes limited degradation in the polymer chains, leading to the formation of low molecular weight species [111].

**Figure 11:**
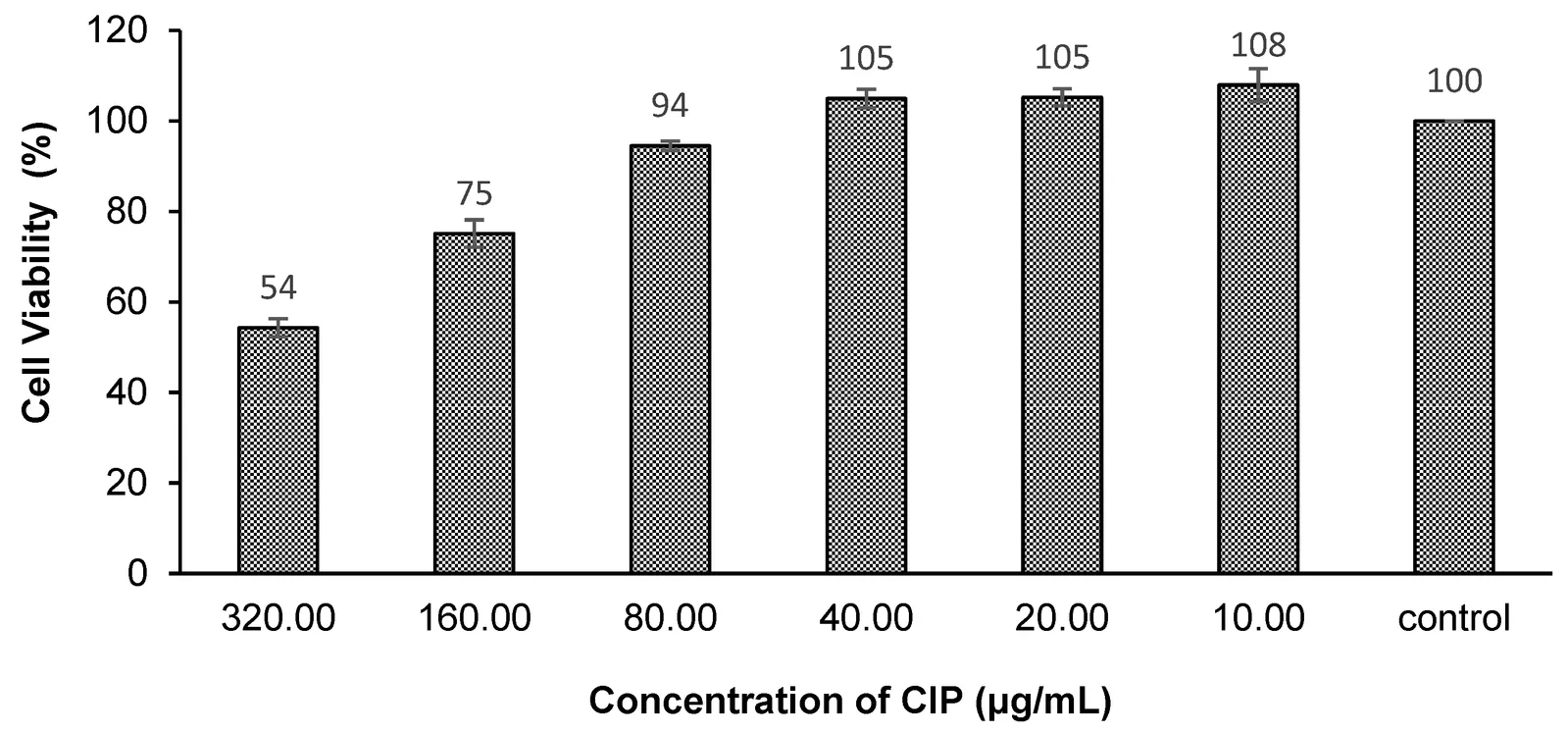
The viability rates (%) of L929 cells in contact with CIP for 24 h.

The cell viability of the PVA/CIP-PVP nanofiber prepared with the addition of 5% CIP decreased to 66.31%. Figure 11 shows the dose-dependent cytotoxic behavior of CIP powder. At low concentrations of CIP in the range of 10–40 µg/mL, cell viability was determined to be 104.92–107.90%. In contrast, increasing the CIP concentration to 160 and 320 µg/mL reduced cell viability to 75.12% and 54.27%, respectively. These results indicate that CIP does not produce a significant cytotoxic effect on fibroblast cells at low concentrations but can become cytotoxic at high local concentrations due to increased cellular stress. The decrease in cell viability of the PVA/CIP-PVP nanofiber, when evaluated together with the dose–response results obtained for free CIP, is not a situation that can be explained solely by the total amount of antibiotic. Indeed, in the 24-h in vitro release experiment, the amount of CIP released from the PVA/CIP-PVP matrix into the medium was determined to be 16.212 µg/mL, a value comparable to the 10–40 µg/mL range that did not negatively affect cell viability in cytotoxicity tests performed with free CIP. Furthermore, the fact that cell viability remained between 104.92 and 107.90% in the presence of 40 µg/mL free CIP indicates that the observed 66.31% viability cannot be explained by a simple “drug overdose” effect. The absence of significant antibacterial activity in the test also supports the idea that CIP is released in a controlled and limited manner from its dense cross-linked network structure. Therefore, the decrease in cell viability in the PVA/CIP-PVP system is more likely related to microenvironmental stress caused by the combination of CIP species localized near the surface and residual GLA, rather than high amounts of CIP release. It has been reported that these local interactions can affect mitochondrial activity and cellular redox balance, thus increasing the formation of reactive oxygen species (ROS) and limiting cell viability [45,112,113].

In addition to residual GLA and surface-bound drug molecules, the direct incorporation of CIP from crushed commercial Cipro^®^ tablets without prior active pharmaceutical ingredient (API) purification represents a methodological limitation that provides a secondary mechanism for this behavior. Commercial tablet formulations contain various insoluble inorganic particulate excipients [114] (such as titanium dioxide and colloidal silica) as well as hydrophobic lubricants (such as magnesium stearate) [115]. Insoluble particulate residues entrapped within the electrospun polymeric matrix can impose localized physical microenvironmental stress and trigger intracellular ROS generation upon direct contact with L929 fibroblasts, thereby contributing to the observed basal cytotoxicity [116,117]. Concurrently, hydrophobic magnesium stearate residues form physical diffusion barriers that retard solvent penetration into the cross-linked matrix, further explaining the severely restricted CIP release (8.51% at 24 h) observed in the PVA/CIP-PVP control group [118,119]. Also, Ciprofloxacin (CIP) can form complexes with metal ions arising from synthesis, particularly through its carboxylate keto groups. This situation may lead to a decrease in the free form of CIP and the formation of metal–CIP complexes [120,121]. The stability of the formed complexes depends on the type of metal ion, pH, and the chemical composition of the environment. Therefore, metal complexation can be considered an additional mechanism that may reduce the diffusion of CIP into the aqueous medium and the overall release rate. This severely restricted release profile is mathematically supported by the kinetic modeling, where the near-zero diffusion exponent (*n* = 0.044) in the Korsmeyer–Peppas model confirms massive physical entrapment within the cross-linked network.

Consequently, unpurified tablet excipients introduce secondary physical and chemical stresses that exacerbate cell viability reduction in the absence of bioactive protective agents.

A significant improvement in cell viability was observed with the inclusion of 3%, 5%, and 7% purple carrot (PCrt) nanofiber concentrate, and the values remained in the range of approximately 75.83–77.00% (Figure 10). This result shows that formulations containing PCrt can partially alleviate cellular stress caused by CIP and GLA residues. The literature reports that purple carrot extracts do not exhibit significant cytotoxic effects on L929 fibroblasts and can maintain cell viability above 75% [122]. The richness of PCrt in chlorogenic acid, ferulic acid derivatives, and cyanidin-based anthocyanins suggests that these components may contribute to limiting cellular oxidative stress through their strong antioxidant properties [123,124]. Therefore, the improvement in cell viability observed in PCrt -containing nanofibers can be explained by, on the one hand, the chemical binding and physical confinement of GLA within the cross-linked network, thereby reducing the accessibility of toxic species, and on the other hand, by the antioxidant effects of phenolic compounds and anthocyanins originating from PCrt.

In conclusion, the findings indicate that the addition of PCrt does not negatively affect the biocompatibility of PVA-PVP-based electrospun nanofiber matrices; on the contrary, it can contribute to reducing cellular stress caused by CIP and GLA residues. The fact that cell viability exceeds 70% in all formulations except PVA/CIP-PVP nanofibers supports the idea that the developed nanofiber structures have a biologically acceptable cell viability profile for use as wound dressings in accordance with ISO 10993-5 criteria.

### 3.8. DPPH Antioxidant Activity Results

**Figure 12:**
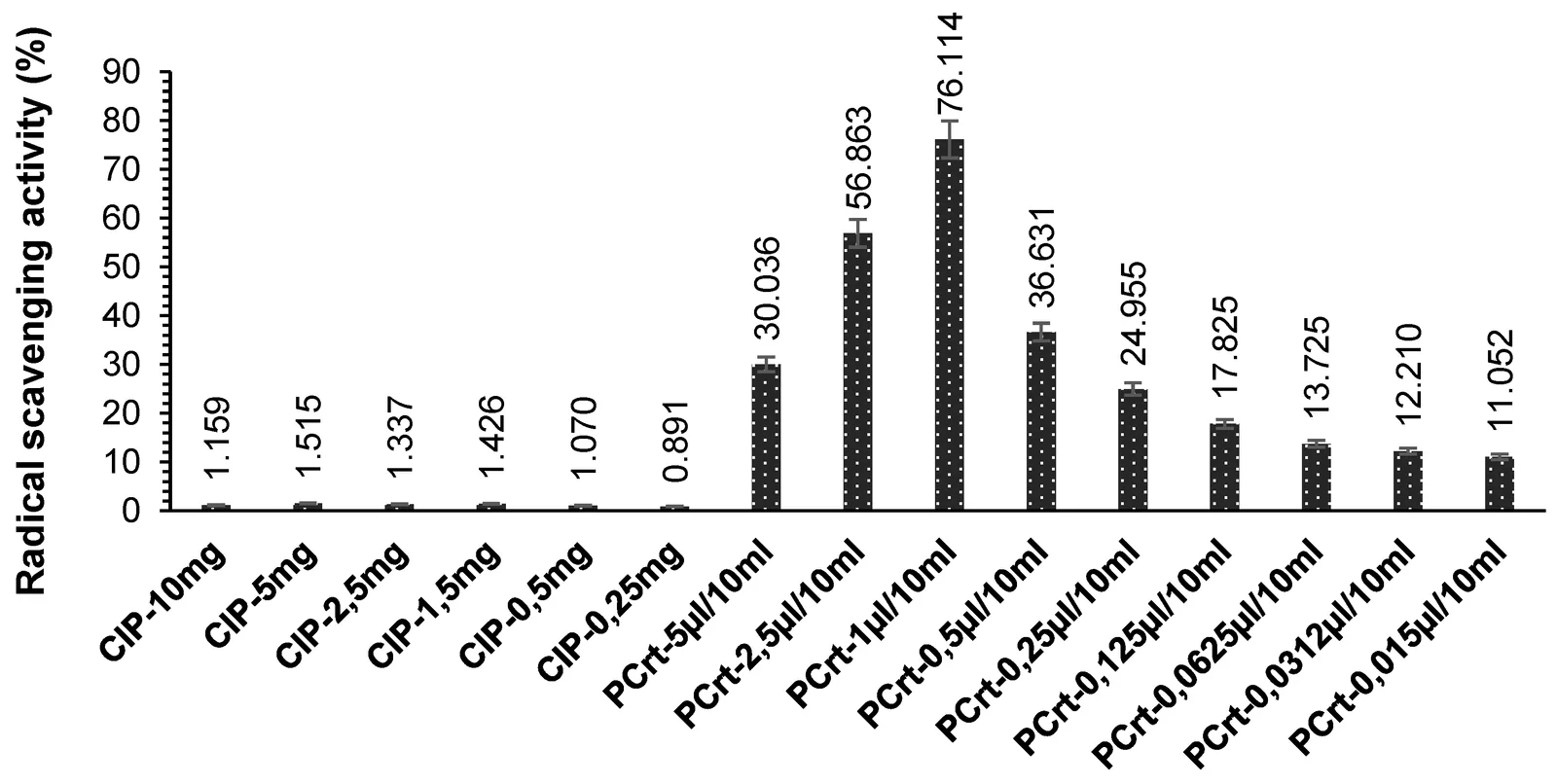
DPPH antioxidant activity of the drug CIP powder and the bioactive substance PCrt concentrate.

In the graphical comparison given in Figure 12, when examining the DPPH antioxidant results of the drug and the bioactive substance, it is seen that the drug CIP, being an antibiotic agent, does not have a radical scavenging effect (in the range of 0.8–1.5%). The bioactive substance PCrt, on the other hand, shows an antioxidant peak of 76.11% at a concentration of 1 µL/10 mL, while this activity decreases significantly to 30.03% when the concentration is increased to 5 µL/10 mL. This situation is attributed to the characteristic properties of anthocyanins and phenolic compounds that are abundant in the structure of PCrt. At high concentrations, these molecules may show a tendency to aggregate among themselves [125,126]. In addition, these phenolic structures may lose their antioxidant properties by undergoing auto-oxidation reactions at high doses and may take on a “pro-oxidant” character [127].

**Figure 13:**
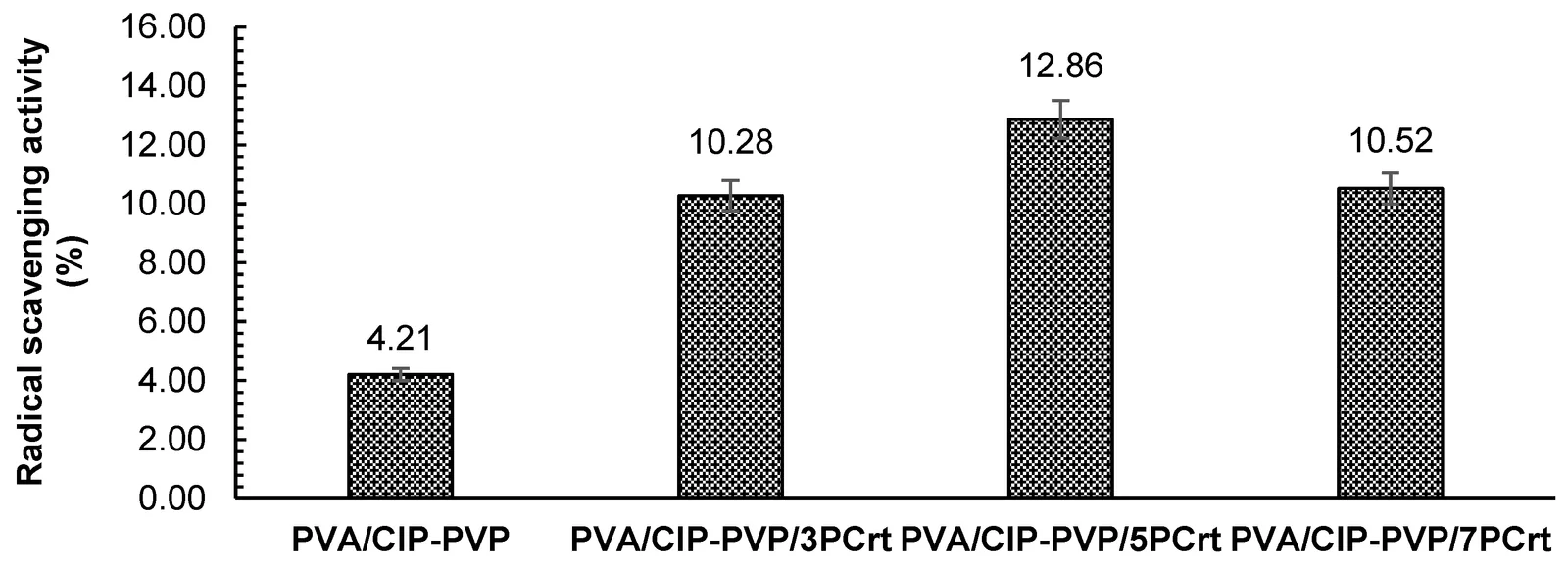
DPPH antioxidant activity of the layered nanofibers.

Figure 13 shows the antioxidant activity when the drug and bioactive agent are encapsulated within the nanofiber. Antioxidant activity, reaching levels of 76% in drug CIP powder and bioactive agent PCrt concentrate, was observed at a maximum of 12.86% (5% PCrt) in the nanofiber form. The incorporation of the active extract into the cross-linked polymer matrix limits the immediate release of bioactive agents and transforms the release profile into a diffusion-controlled delayed response [126,128]. In this study, this altered the immediate accessibility of the active components within the nanofiber to external DPPH radicals, extending the instantaneous chemical reaction over time.

The lower in vitro DPPH scavenging activity of PCrt-loaded nanofibers compared to free extract is attributed to structural entrapment and diffusion resistance within the polymeric matrix. While free extract immediately dissolves and reacts, nanofiber-embedded phenolics are constrained by physical entrapment and non-covalent interactions, restricting rapid diffusion into the assay medium. This diffusion resistance is heavily corroborated by the in vitro release kinetics, wherein the highly negative polymer relaxation constant (*k*_2_) in the Peppas–Sahlin model and the restricted plateau in the percentage release graph (limiting proportional release to ~22%) prove that the entangled polymer matrix structurally prevents the rapid outward diffusion of phenolic compounds.

Clinically, this limited immediate accessibility prevents rapid burst release and wash-out by exudates, providing sustained, long-term antioxidant protection to continuously neutralize ROS at the wound site. Future translational in vivo studies will evaluate how this controlled-release profile modulates tissue ROS levels during active healing. It is thought that the low DPPH scavenging activity observed in PCrt-loaded nanofibers may be due to structural properties that limit the diffusion of active ingredients through the polymeric matrix, rather than an actual loss in the biological activity of the extract, which may provide a functional advantage in terms of controlled release, it is believed that the DPPH scavenging activity observed in PCrt-loaded nanofibers is not a real loss in the biological activity of the extract. This limited accessibility can contribute to a more controlled and sustainable release of active ingredients from the matrix by preventing the sudden and rapid release of the extract. Therefore, the low initial antioxidant activity observed in the nanofiber system can be evaluated not only as a loss of activity, but also as a reflection of controlled release behavior.

## 4. Conclusions

In this study, GLA-crosslinked bilayer PVA/CIP-PVP/PCrt nanofiber matrices were produced for a highly functional, biocompatible, and synergistic treatment system for wound dressing applications. Diameter analyses of the nanofibers showed that the addition of 3% PCrt extract increased the solution conductivity and net charge density, reducing the fiber diameter to the finest level (302.86 ± 41.3 nm), but at 5% and 7% concentrations, viscosity and chain entanglement effects prevailed, triggering fiber thickening.

A critical mechanistic paradox was uncovered between swelling capacity and drug release: although the abundant hydrophilic -OH groups of PCrt drove massive macroscopic water uptake (up to 707.14%), quantitative kinetic modeling proved that the active agents remained strictly confined. The highly negative polymer relaxation constant (*k*_2_) in the Peppas–Sahlin model and Quasi-Fickian diffusion exponents (*n* < 0.45) mathematically confirmed that intense hydrogen bonding and GLA cross-links physically locked the matrix. This structural confinement restricted the proportional release efficiency to ~22.8% at high loading and severely limited baseline CIP release to 8.51%.

In pH sensitivity tests, the fact that the fibers, which turned pink due to the formation of flavonoid cations during the acid-catalyzed cross-linking step, remained completely white in subsequent washing and pH steps proved that hydrophilic anthocyanins leached into the washing water. The prominent carbonyl peaks observed at 2860 cm^−1^ and 1734 cm^−1^ in the FTIR spectrum confirmed the presence of unreacted free GLA residues in the matrix despite the washing processes.

Indeed, the 100% antibacterial activity observed in the PVA-PVP control group and the 100% bactericidal recovery in the PCrt-loaded groups appear to be primarily triggered by the mobilization of these leached free GLA residues. Although the antioxidant phenolic compounds in PCrt may help mitigate local chemical stress and contribute to maintaining fibroblast viability within the 75–77% range, the basal cytotoxicity observed in the PVA-PVP control nanofiber may warrant further consideration regarding its potential impact on the biological safety of the developed wound dressing.

In conclusion, it is thought that the developed layered nanofibers, thanks to their strong antibacterial properties and high swelling capacity, can contribute to maintaining a moist environment in the wound area and preventing microbial infections; and that other nanofibers excluding the PVA-CIP/PVP nanofiber, which were found suitable in terms of cell viability, may find potential applications in various biomedical applications, especially in wound dressings.

***Translational and Clinical Outlook***

While the developed PCrt-loaded bilayer nanofibrous dressing exhibits promising multifunctional properties for wound care, several technological and regulatory challenges must be addressed for successful clinical translation:

(i) Transitioning from bench-scale single-needle electrospinning to industrial needleless or multi-jet electrospinning requires precise optimization to ensure batch-to-batch reproducibility, uniform fiber thickness, and consistent bioactive loading.

(ii) Conventional high-temperature or radiation-based sterilization methods risk degrading thermosensitive anthocyanin chromophores and altering polymer network integrity. Implementing non-thermal sterilization techniques (such as low-temperature ethylene oxide or supercritical CO_2_ processing) combined with moisture- and light-barrier packaging will be essential to preserve bioactive stability during storage.

(iii) In heavily exudative chronic wounds, excessive swelling may compromise structural robustness. Optimizing cross-linking density or applying hydrophobic outer barrier layers will mitigate premature mechanical weakening and control wash-out kinetics.

(iv) Translational approval demands strict compliance with ISO 10993 standards. Future studies will focus on transitioning toward green, non-toxic cross-linking agents (e.g., citric acid) and conducting comprehensive *in vivo* full-thickness wound healing models to fully evaluate long-term biocompatibility, tissue regeneration, and therapeutic efficacy.

(v) In chronic wounds, pH, ROS level, enzyme activity, and exudate composition differ from healthy skin. Therefore, testing the system not only in buffer solutions, but also in models that mimic the physiological and chronic wound microenvironment will allow a more accurate prediction of actual clinical performance.

(vi) The shelf life of the nanofiber coating under real storage conditions must be determined. Changes in the packaging’s moisture, oxygen, and light permeability, as well as its mechanical properties and bioactive substance content, should be monitored together.

## Figures and Tables

**Table 1:** Composition and sample codes of the produced nanofibers.

Sample Code	First Layer % (*w*/*w*)	Second Layer % (*w*/*w*)
PVA-PVP	100 PVA	100PVP
PVA/CIP-PVP	95PVA/5CIP	100PVP
PVA/CIP-PVP/3PCrt		97PVP/3PCrt
PVA/CIP-PVP/5PCrt		95PVP/5PCrt
PVA/CIP-PVP/7PCrt		93PVP/7PCrt

**Table 2:** Concentrations of CIP and PCrt used as standards.

Active Ingredient	Concentrations Used as Standards						
CIP	100 µg/mL	50 µg/mL	25 µg/mL	5 µg/mL	2.5 µg/mL	1 µg/mL	0.5 µg/mL
PCrt	1 µL/10 mL	0.5 µL/10 mL	0.25 µL/10 mL	0.125 µL/10 mL	0.0625 µL/10 mL	0.03125 µL/10 mL	

**Table 3:** Kinetic parameters and statistical evaluation of in vitro release profiles from the cross-linked bilayer nanofiber matrices.

Release Model	Parameter	PVA/CIP-PVP	PVA/CIP-PVP/3PCrt	PVA/CIP-PVP/5PCrt	PVA/CIP-PVP/7PCrt
Zero-order	*k*_0_	0.560	2.776	1.465	1.451
	$R_{\mathrm{adj}}^{2}$	−274.134	−30.433	−17.230	−14.275
	RMSE	6.314	28.490	14.923	14.381
First-order	*k*_1_	0.006	0.084	0.020	0.020
	$R_{\mathrm{adj}}^{2}$	−264.801	−18.317	−14.930	−12.317
	RMSE	6.206	22.334	13.951	13.428
Higuchi	*k_H_*	2.849	13.797	7.273	7.150
	$R_{\mathrm{adj}}^{2}$	−94.261	−8.334	−4.295	−3.249
	RMSE	3.715	15.525	8.043	7.584
Korsmeyer–Peppas	*k_KP_*	7.510	31.827	16.153	15.406
	*n*	0.044	0.114	0.135	0.150
	$R_{\mathrm{adj}}^{2}$	0.853	0.744	0.596	0.649
	RMSE	0.146	2.569	2.222	2.180
Peppas–Sahlin	*k*_1_	10.773	36.369	16.378	15.207
	*k*_2_	−3.397	−7.454	−2.727	−2.352
	$R_{\mathrm{adj}}^{2}$	0.895	0.915	0.901	0.929
	RMSE	0.124	1.480	1.101	0.982

$R_{\mathrm{adj}}^{2}$: Adjusted coefficient of determination; RMSE: Root mean square error; *k*_0_: Zero-order release constant; *k*_1_ (First-order): First-order release constant; *k_H_*: Higuchi release constant; *k_KP_*: Korsmeyer–Peppas release constant; *n*: Diffusional exponent indicating the release mechanism; *k*_1_ and *k*_2_ (Peppas–Sahlin): Fickian diffusion constant and polymer relaxation constant, respectively.

## Data Availability

The raw data supporting the conclusions of this article will be made available by the authors on request.

## Abbreviations

The following abbreviations are used in this manuscript:

PVA	Polyvinyl alcohol
PVP	Polyvinylpyrrolidone
CIP	Ciprofloxacin
PCrt	Purple carrot
GLA	Glutaraldehyde
HCl	Hydrochloric acid
PBS	Phosphate-buffered saline
SEM	Scanning electron microscopy
FTIR	Fourier-transform infrared spectroscopy
DPPH	2,2-diphenyl-1-picrylhydrazyl
CCK-8	Cell Counting Kit-8
WST-8	Water-soluble tetrazolium salt-8
ROS	Reactive oxygen species
ISO	International Organization for Standardization
OD	Optical density
UV	Ultraviolet
AATCC	American Association of Textile Chemists and Colorists
SCDLP	Soybean-Casein Digest Lecithin Polysorbate
NA	Nutrient Agar
NB	Nutrient Broth
